# Obstetric Complications and Pregnancy Outcomes in Cancer Survivors: A Systematic Review and Meta-Analysis

**DOI:** 10.3390/cancers17243924

**Published:** 2025-12-08

**Authors:** Charmaine Yong Ching Lee, Isaac Yongjie Sim, Chen Ee Low, Tessa Ying Zhen Tan, Abdul Fattah Lee Abdul Aziz, Zhongwei Huang, Jeremy Chee Seong Tey, Ainsley Ryan Yan Bin Lee

**Affiliations:** 1 Department of Medicine, Yong Loo Lin School of Medicine, National University of Singapore, Singapore 117597, Singapore; e0771305@u.nus.edu (C.Y.C.L.); e0905183@u.nus.edu (I.Y.S.); cheneelow@u.nus.edu (C.E.L.); tantessa@u.nus.edu (T.Y.Z.T.); abdulfattahlee@u.nus.edu (A.F.L.A.A.); jeremy_tey@nuhs.edu.sg (J.C.S.T.); 2 Division of Reproductive Endocrinology & Infertility, Department of Obstetrics & Gynaecology, National University Hospital, Singapore 119074, Singapore; obgzwh@nus.edu.sg; 3 NUS Bia-Echo Asia Centre for Reproductive Longevity and Equality (ACRLE), National University of Singapore, Singapore 117456, Singapore; 4 Department of Radiation Oncology, National University Cancer Institute, Singapore 119074, Singapore

**Keywords:** obstetric outcomes, pregnancy, cancer survivorship, childhood cancer

## Abstract

**Background:** With advances in cancer diagnosis and therapy, survival after childhood and young-adult cancers has improved markedly. As survivorship extends, understanding long-term health sequelae, including obstetric outcomes, has become increasingly important. However, the reproductive safety of pregnancy following cancer remains insufficiently characterized. This systematic review and meta-analysis aims to provide a comprehensive evaluation of obstetric outcomes following pregnancy in survivors of childhood and young-adult cancers. **Methods:** We conducted a systematic review and meta-analysis (PROSPERO: CRD42024573707) of PubMed, Embase, and Cochrane databases to identify controlled studies assessing obstetric complications among female cancer survivors, published between 1 January 2000 and 31 June 2024. Random effects meta-analyses were used to estimate pooled risk ratios (RRs) with 95% confidence intervals (CIs). Heterogeneity, subgroup analyses, and meta-regression were performed to identify sources of variation. **Results:** Of 6032 records screened, 16 studies involving 89,123 survivors and 21,569,191 controls were included. Cancer survivorship was associated with higher risks of preeclampsia (RR 1.37, 95% CI 1.17–1.62), gestational diabetes (RR 1.29, 95% CI 1.05–1.59), and miscarriage (RR 1.16, 95% CI 1.01–1.35), but not with anemia in pregnancy (RR 1.16, 95% CI 0.98–1.39) or hypertensive disorders (RR 1.21, 95% CI 0.99–1.49). Cancer type emerged as a potential prognostic factor for preeclampsia. **Conclusions:** Female cancer survivors are at significantly increased risk of major obstetric complications, underscoring the need for anticipatory preconception counselling and enhanced antenatal surveillance. Future research should delineate cancer- and treatment-specific risks to inform precision reproductive care in this growing survivorship population.

## Simple Summary

With advances in cancer diagnosis and therapy, survival after childhood and young adult cancers has improved markedly. Obstetric outcomes have become an increasingly important part of these survivors’ long-term health sequelae. Overall, we found that women who previously had cancer were more likely to experience certain pregnancy complications compared with women who never had cancer. These included a higher chance of developing preeclampsia, gestational diabetes, and miscarriage. However, cancer survivors did not appear to have higher risks of anemia in pregnancy or general high blood pressure outside of preeclampsia. The type of cancer someone had may also influence their pregnancy risks, although the evidence is still mixed and varies from study to study. These findings highlight that cancer survivors may be at increased risk of certain obstetric complications and may benefit from closer monitoring, pre-pregnancy counselling, and personalized care during pregnancy. Further research is needed to understand how specific cancers and treatments affect reproductive health to guide obstetric care in cancer survivors.

## 1. Introduction

Revolutionary advances in cancer diagnosis, multimodal therapy, and supportive care have transformed and vastly improved the prognosis of childhood malignancies, with 5-year survival rates now exceeding 80% in high-income countries [1]. As a growing population of children and young adults survives their disease, the focus of oncology has necessarily broadened from developing a cure alone to encompass survivorship and long-term quality of life [2,3,4]. The long-term medical, psychological, and social sequelae of cancer are increasingly recognized as integral components of post-treatment care [5,6,7,8]. Among these, the ability to conceive and bear children has emerged as a defining contributor to emotional well-being, life satisfaction, and identity among survivors, particularly as many individuals in long-term remission reach reproductive age and begin to consider family building as an essential marker of recovery and normalcy [9]. Despite these gains, the very therapies that enable survival, cytotoxic chemotherapy, radiotherapy, and surgery, may have enduring and incompletely understood effects on multiple physiological systems, including reproductive health [10,11,12], with potential consequences that may only manifest years after treatment completion, well beyond the typical surveillance period for oncologic relapse. Gonadal damage, uterine vascular impairment, and hypothalamic–pituitary axis dysfunction have all been implicated in mediating adverse reproductive outcomes. Yet the extent to which these biological insults translate into clinically meaningful obstetric complications remains uncertain. Understanding the safety and outcomes of pregnancy among female cancer survivors therefore represents not only a scientific imperative but also a cornerstone of comprehensive survivorship care.

Several population-based studies have investigated obstetric outcomes in cancer survivors, evaluating risks of preeclampsia, gestational diabetes, miscarriage, and other complications. However, findings have been heterogeneous. While Kao et al. [13] and Rubens et al. [14] reported an elevated risk of obstetric complications among survivors, Winther et al. [15] found pregnancy outcomes comparable to those of the general population. These inconsistencies in the available literature likely reflect differences in study design, cancer types, treatment exposures, and follow-up durations, underscoring the need for a rigorous synthesis of available evidence.

This systematic review and meta-analysis aims to provide a comprehensive evaluation of obstetric outcomes following pregnancy in survivors of childhood and young-adult cancers, with the overarching goal of supporting informed reproductive decision making among survivors and their healthcare providers. By integrating data across diverse populations and treatment contexts, we seek to clarify the magnitude and nature of obstetric risks, identify vulnerable subgroups, and inform evidence-based counselling and management for women contemplating pregnancy after cancer. To our knowledge, this is the first systematic review and meta-analysis to quantitatively delineate the impact of prior cancer and its treatment on obstetric complications among survivors, thereby addressing a critical gap in the literature and advancing the agenda of holistic, life-course cancer care.

## 2. Methods

We reported our systematic review according to the Preferred Reporting Items for Systematic Reviews and Meta-Analyses (PRISMA) guidelines. This protocol was registered on the International Prospective Register of Systematic Reviews (PROSPERO) (Reference: CRD42024573707).

### 2.1. Search Strategy

A literature search was performed in PubMed, Embase and Cochrane. Our search strategy combined terms for pregnancy outcome, childhood cancer survivor, and young adult cancer survivors. The database-controlled vocabulary was used to search subject headings. We used a spectrum of synonyms with appropriate truncations to search titles, abstracts, and author keywords. The search strategy was repeated across the databases. Examples of the search strategies for PubMed and Embase are found in Supplementary Table S1.

### 2.2. Inclusion and Exclusion Criteria

**Figure 1:**
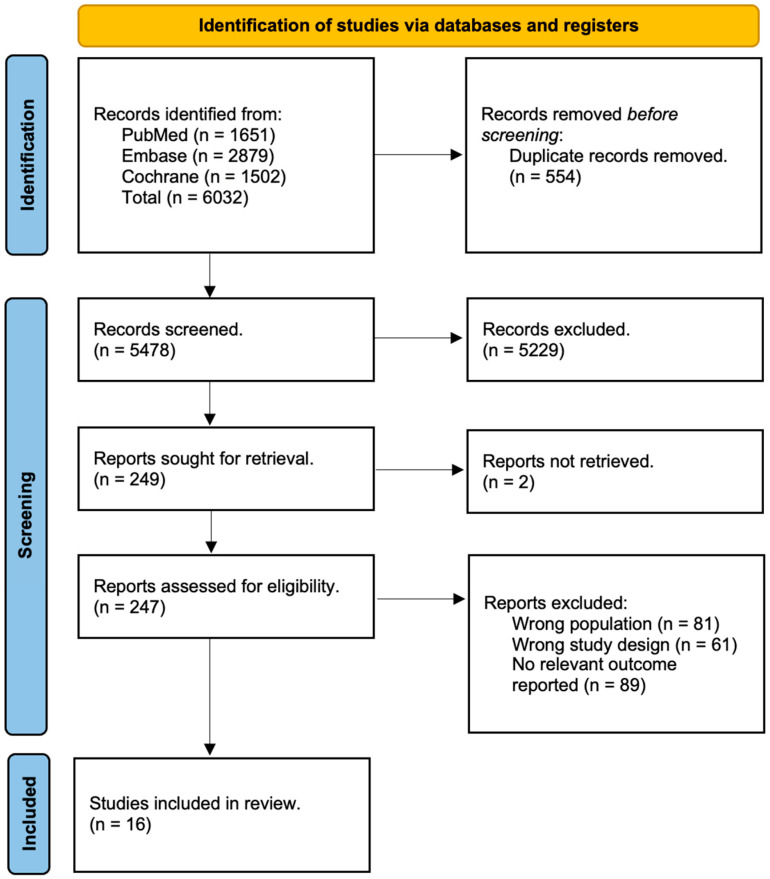
PRISMA flowchart.

Three reviewers independently screened titles and abstracts of all studies for eligibility. The full texts of studies assessed as ‘relevant’ or ‘unclear’ were then independently assessed by a fourth reviewer. Any discrepancies between authors during title and abstract screening or full-text eligibility assessment were subsequently resolved. All peer-reviewed English-language studies published from 1 January 2000 to 31 June 2024 that evaluated obstetric outcomes following pregnancy in childhood and young adult cancer survivors were included. Non-empirical studies, grey literature, and studies without a control arm or abstract were excluded. The selection process is shown in Figure 1.

### 2.3. Data Extraction

Two reviewers independently performed the extraction with quality checking at the end. Subject matter information included the aim of the study, demographics, cancer type, the age of diagnosis and delivery, the method of fertilization if reported, smoking history, the characteristics of the control group, and the main outcomes of the study, specifically regarding preeclampsia, gestational diabetes, anemia in pregnancy, hypertension in pregnancy, and miscarriage. Preeclampsia is defined as hypertension occurring after twenty weeks of gestation with the presence of end organ damage and proteinuria [16]. Gestational diabetes refers to hyperglycaemia with onset during pregnancy [17]. Anemia in pregnancy is defined by reduction in the concentration of erythrocytes or hemoglobin in blood [18]. Hypertension in pregnancy is defined by systolic blood pressure above 140 mmHg or diastolic blood pressure above 90 mmHg on 2 separate occasions at least 4 h apart after 20 weeks of pregnancy with a normal baseline blood pressure [19]. Miscarriage, otherwise known as spontaneous abortion, is defined as the loss of a pregnancy less than 20 weeks of gestation [20]. The number of participants and controls at risk and the number of events regarding the specific outcomes were extracted.

### 2.4. Statistical Analysis

We conducted all analyses on R (version 4.1.0) using the *meta* and *metafor* packages. A two-sided *p* value of <0.05 was considered statistically significant. Studies were pooled for meta-analyses using the relative risk of the obstetric outcomes (measured using risk ratios (RR) compared to controls). Sensitivity analysis was conducted using the identification and exclusion of potential outliers and the leave-one-out analysis. Between-study heterogeneity was represented using I2 and τ2 statistics. An I2 of <30% demonstrated low heterogeneity between studies, one of 30% to 60% revealed moderate heterogeneity, and a value > 60% showed substantial heterogeneity. We performed subgroup analyses and meta-regression to determine if any key categorical and hierarchical variables influenced the results. We assessed for publication bias quantitatively using Egger’s test [21]. Visual inspection for funnel plot asymmetry was used for qualitative publication bias [22]. If we suspected publication bias, sensitivity analysis was conducted using the trim-and-fill method (R0 estimator, fixed random effects models) to estimate the pooled effect size after imputing potential studies [23]. If publication bias was absent, this was because of a normal distribution of effect sizes around the centre of the funnel plot [22].

### 2.5. Risk of Bias

Two independent reviewers assessed for the methodological quality and risk of bias of the included studies using the Joanna Brigg’s Institute (JBI) Critical Appraisal tool [24]. Any discrepancies were resolved by a third reviewer. Certainty of evidence was assessed using the GRADE framework [25].

## 3. Results

A total of 16 studies were included from 6032 records (Figure 1).

A further 6016 studies were excluded after removing studies with irrelevant study designs, populations, outcomes, and duplicates. A total of 89,123 cancer survivors and 21,569,191 controls were investigated. Three studies were from Asia [13,26,27], eight studies were from Europe [15,28,29,30,31,32,33,34], four studies were from North America [14,35,36,37] and one study was from Oceania [38]. Twelve studies reported on preeclampsia [13,14,26,28,29,32,33,34,35,36,37,38], nine studies reported on gestational diabetes [13,14,26,28,29,33,36,37,38], six studies reported on maternal anemia [13,26,28,36,37,38], five studies reported on hypertensive disorders of pregnancy [14,27,28,33,35], four studies reported on miscarriage [15,30,31,32], and one study reported on thromboembolic events [14] and maternal death [14]. Ten studies had matched controls [13,26,28,30,31,32,34,35,36,38], three studies had population controls [27,35,37], and three studies had sibling controls [15,29,33]. One study had both population and matched controls [35]. The main characteristics of the included studies can be found in Table 1.

### 3.1. Preeclampsia

**Figure 2:**
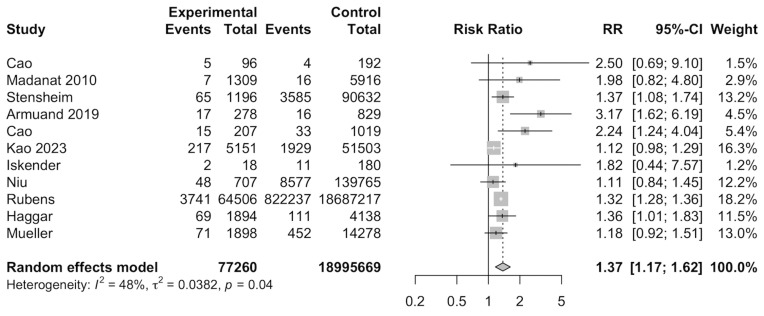
Forest plot for risk of preeclampsia among cancer survivors [13,14,26,28,29,32,33,34,36,37,38].

In total, 11 studies [13,14,26,28,29,32,33,34,36,37,38] were included for meta-analysis to evaluate the RR of preeclampsia among cancer survivors. Meta-analysis of the 11 studies indicated that female cancer survivors had a significantly increased risk of developing preeclampsia (RR: 1.37, 95%CI: 1.17–1.62) (Figure 2). A total of 19,072,929 individuals were investigated and 841,228 developed preeclampsia. Subgroup analyses found that categorical variables such as type of control, region of study, type of cancer, age of diagnosis, age of delivery, and smoking were insignificant (Supplementary Table S2). Meta-regression was also insignificant (Supplementary Table S4).

### 3.2. Gestational Diabetes Mellitus

**Figure 3:**
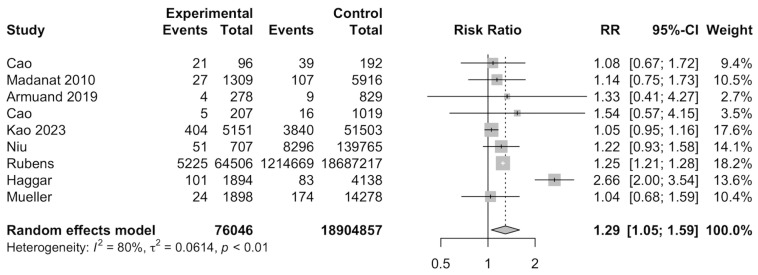
Forest plot for risk of gestational diabetes among cancer survivors [13,14,26,28,33,36,37,38].

Nine studies [13,14,26,28,29,33,36,37,38] were included for meta-analysis to evaluate the RR of gestational diabetes among cancer survivors. Meta-analysis of the nine studies indicated that cancer survivors had a significantly increased risk of developing gestational diabetes (RR: 1.29, 95%CI: 1.05–1.59) (Figure 3). A total of 18,980,903 individuals were investigated and 1233,095 developed gestational diabetes. Subgroup analyses found that North American studies showed a significantly increased risk ratio for developing gestational diabetes (RR: 1.25, 95%CI: 1.21–1.28), when compared to European countries (RR: 1.21, 95%CI: 0.84–1.74) and Asian countries (RR: 1.05, 95%CI: 0.96–1.16). Other categorical variables, such as type of control, type of cancer, age of diagnosis, age of delivery, and smoking, were insignificant (Supplementary Table S3). Meta-regression was also insignificant (Supplementary Table S4).

### 3.3. Anemia in Pregnancy

Six studies [13,26,28,36,37,38] were included in meta-analyses to evaluate the RR of anemia in pregnancy among cancer survivors. A total of 220,729 individuals were investigated and 17,508 developed anemia in pregnancy. Meta-analysis of the six studies indicated that cancer survivors had no significant risk of developing anemia in pregnancy (RR: 1.16, 95%CI: 0.98–1.39) (Supplementary Figure S1).

### 3.4. Hypertension in Pregnancy

Four studies [14,28,33,35] were included in the meta-analyses to evaluate the RR of hypertension in pregnancy among cancer survivors. A total of 21,231,233 individuals were investigated and 931,799 developed hypertension in pregnancy. Meta-analysis of the four studies indicated that cancer survivors had no significant risk of developing hypertension in pregnancy (RR: 1.21, 95%CI: 0.99–1.49) (Supplementary Figure S2).

### 3.5. Miscarriage

Four studies [15,30,31,32] were included in the meta-analyses to evaluate the RR of miscarriage among cancer survivors. A total of 8523 individuals were investigated and 746 individuals had miscarriages. Meta-analysis of the four studies indicated that cancer survivors had a significantly increased risk of miscarriage (RR: 1.16, 95%CI: 1.01–1.35) (Supplementary Figure S3).

### 3.6. Systematic Review of Treatment Modality

#### 3.6.1. Preeclampsia

Three studies were investigated. All three studies [13,26,32] found no significant risk of developing preeclampsia with any treatment modality, including radiotherapy [13,26], chemotherapy with radiotherapy [13], and stem cell transplantation [32] (Supplementary Table S5).

#### 3.6.2. Gestational Diabetes

Four studies [13,26,36,38] were investigated. Only one study found a significantly increased risk for developing gestational diabetes for those treated with both radiotherapy and chemotherapy [38] (Supplementary Table S5).

#### 3.6.3. Anemia in Pregnancy

Three studies were investigated. All three studies [13,26,36] found no significant risk of developing anemia in pregnancy with any treatment modality, including radiotherapy [13,26,36] and chemotherapy [13,36] (Supplementary Table S5).

#### 3.6.4. Miscarriage

Three studies [15,30,32] were investigated. One study found a significantly increased risk of miscarriage among those treated with radiotherapy, especially when applied to the ovaries, uterus, and pituitary gland [15]. The other two studies found no significant risk of miscarriage among those treated with radiotherapy [30], chemotherapy [30] and stem cell transplantation [32] (Supplementary Table S5).

### 3.7. Systematic Review of Cancer Type

#### 3.7.1. Preeclampsia

Four studies [26,28,29,36] were investigated. Two out of four studies found a significantly increased risk of preeclampsia in survivors of hematological malignancies [28], neuroendocrine tumours [28], and colorectal cancer [29]. The other two studies found no significant increase in risk of preeclampsia among survivors of thyroid cancer [26] and genital tract carcinoma [36] (Supplementary Table S6).

#### 3.7.2. Gestational Diabetes

Four studies [26,28,36,38] were investigated. Two studies found a significantly increased risk of developing gestational diabetes among survivors of bone cancer [36,38]. Carcinoma and central nervous system tumours were reported in two studies, with one showing that survivors had an increased risk of gestational diabetes [38], while the other study found no significant risk [36] (Supplementary Table S6).

#### 3.7.3. Anemia in Pregnancy

Two studies [26,36] were investigated. One study investigated central nervous tumours and found a significantly increased risk of anemia in pregnancy among survivors [36]. Both studies investigated thyroid cancer [26,36] and one found a significantly increased risk of anemia in pregnancy [26], while the other showed no significant risk [36] (Supplementary Table S6).

#### 3.7.4. Miscarriage

Three studies [15,26,30] were investigated. Leukemia was investigated in two studies [15,30], with one showing a significantly increased risk of miscarriage [30], while the other showed no significant risk [15]. Renal tumours were investigated in two studies [15,30], with one showing a significantly increased risk of miscarriage [15], while the other showed no significant risk [30]. Germ cell tumours were investigated in two studies [15,30], with one showing significantly increased risk of miscarriage [15], while the other showed no significant risk [30] (Supplementary Table S6).

#### 3.7.5. Risk of Bias, Publication Bias and Certainty of Evidence

Assessments of risk of bias and certainty of evidence are presented in Supplementary Tables S7 and S8. Overall, there were no studies with a high risk of bias. There was moderate certainty of evidence as assessed using the GRADE framework. Publication bias was assessed qualitatively and quantitatively by funnel plot inspection, trim-and-fill analysis and Egger’s test for the outcomes of preeclampsia and gestational diabetes, which had 10 or more studies valid for meta-analysis (Supplementary Figures S4–S9). Overall, no significant publication bias was detected. Sensitivity analysis was performed using leave-one-out and outlier analysis, which yielded no significant changes in effect size and outcomes (Supplementary Figures S10–S13).

## 4. Discussion

This systematic review and meta-analysis provides the most comprehensive synthesis to date of obstetric outcomes among female cancer survivors. We found that these cancer survivors were at a significantly higher risk of preeclampsia, gestational diabetes mellitus, and miscarriage compared with women without a history of cancer. Subgroup analyses further suggested that cancer type may act as a prognostic factor for preeclampsia. These findings corroborate previous epidemiological observations on the long-term systemic effects of cancer and its treatment, while adding quantitative evidence that underscores their clinical relevance. Nevertheless, considerable heterogeneity across studies—reflecting variations in cancer types, treatment exposures, and demographic profiles—highlights the complexity [39,40] of disentangling the multifactorial determinants of adverse obstetric outcomes. To our knowledge, this is the first systematic review and meta-analysis to delineate, in aggregate, the impact of prior cancer on maternal and obstetric complications.

### 4.1. Preeclampsia

Our analyses identified a significantly increased risk of preeclampsia among female cancer survivors. Although the mechanistic basis for this association remains incompletely understood, several immunological and vascular pathways provide plausible explanations, particularly involving persistent immune dysregulation secondary to prior malignant disease and treatment exposure. Elevated levels of CD4^+^CD25^+^ regulatory T (Treg) cells, which were first reported in patients with lung and ovarian cancers by Woo et al. [41,42] following their discovery in 1995 [43], have been found to be implicated in tumour immune evasion [44,45,46,47,48]. Cancer survivors may exhibit persistently elevated or memory Treg populations even after disease remission. In pregnancy, decidual natural killer (dNK) cell-mediated angiogenesis is tightly regulated by cytokines such as Treg-derived transforming growth factor β1 (TGFb1) [49]. Excess TGFβ1 disrupts the balance of dNK subpopulations, suppresses vascular endothelial growth factor (VEGF) expression, and impairs spiral artery remodelling, all of which are key events in the pathogenesis of placental ischemia and, subsequently, preeclampsia [49]. Zhang et al. demonstrated that preeclamptic deciduae contained markedly higher concentrations of TGFβ1 than normal term samples (4557 pg/mL vs. 1974 pg/mL; *p* < 0.05) [49], suggesting a possible mechanistic link between elevated Treg activity and preeclampsia risk in cancer survivors.

In addition, established cardiovascular comorbidities may contribute to this association, acting synergistically with immune-mediated mechanisms to heighten vascular vulnerability during pregnancy. Hypertension, which was found to be present in up to 38% of cancer patients, is among the most prevalent chronic conditions in this population [50]. Existing epidemiological evidence suggests a bidirectional relationship between hypertension and certain cancers, including renal cell carcinoma [51,52] and oesophageal cancer [53]. Moreover, several anticancer agents, particularly VEGF pathway inhibitors, are associated with new-onset hypertension or its exacerbation of [54]. As preexisting hypertension is a well-established risk factor for preeclampsia [52], treatment-induced or pre-morbid hypertension in survivors may partially account for the observed excess risk. Advanced maternal age is an additional confounder, with nearly half of pregnant cancer survivors aged ≥ 35 years [55]. Advanced maternal age, defined as being over 35 at the time of delivery [56], is widely recognized as a risk factor for preeclampsia. Numerous studies have shown a correlation between advanced maternal age and an increased risk of preeclampsia, which may partly account for the elevated risk observed in these cancer survivors [56].

### 4.2. Gestational Diabetes Mellitus

Risk factors for gestational diabetes mellitus include that of advanced maternal age, pre-existing diabetes mellitus and obesity, all of which appear to be over-represented in subsets of cancer survivors. In normal pregnancy, insulin sensitivity fluctuates, with higher sensitivity during early pregnancy and decreased sensitivity during late pregnancy [57]. This is due to the increase in placental hormones such as progesterone and human placental lactogen, which promotes a mild state of insulin resistance [57]. Our study has shown that there is a significantly increased risk of developing gestational diabetes among cancer survivors. Cancer and its treatments may result in prolonged stress even after the disease is gone [58]. Potential reasons for this include the fear of potential recurrence of disease as well as possible hereditary cancer risk in their offspring. Prolonged stress results in increased cortisol release, which plays a key role in the development of obesity. Increased cortisol leads to higher visceral fat deposition, insulin resistance and potentially increases the desire for food rich in fat and sucrose [59], contributing to an adverse metabolic profile that precedes pregnancy. These factors, combined with decreased physical activity secondary to cancer related fatigue which persists beyond treatment [7,60], precipitate the development of obesity and insulin resistance, thereby compounding baseline metabolic risk. Additionally, cancer treatments such as chemotherapy [61] and radiotherapy [38] have also been proven to induce insulin resistance. This is further compounded by the use of glucocorticoids, which are commonly prescribed to alleviate the adverse side effects of chemotherapy, and are also known to cause insulin resistance and hyperglycemia [62], resulting in effects similar to those of prolonged stress. These factors, coupled with pregnancy, result in an increased state of insulin resistance, which triggers beta cell dysfunction. Hyperglycemia subsequently develops as a result of insulin resistance and can cause glucotoxicity when prolonged, perpetuating a vicious cycle which ultimately increases the risk of developing gestational diabetes in cancer survivors [57].

### 4.3. Miscarriage

Our results have shown a significantly increased risk of miscarriage in cancer survivors. The precise etiology of miscarriage in this population is varied, ranging from fetal chromosomal abnormalities to inflammatory and immunologic dysregulation [63]. Although there is limited existing literature explaining this relationship, we postulate the following reasons to draw the link between the two conditions. Firstly, cancer survivors tend to have more advanced maternal age at time of conception [55]. Yasuoka et al. found 48.3% of pregnant cancer survivors to be above 35 years of age [55]. It is widely accepted that female fertility declines gradually after the age of 20, and precipitously after the age of 35 [64]. A decrease in oocyte quality alongside lower progesterone concentration and a less receptive endometrium may lead to increased risk of miscarriage in waning reproductive years [64]. Secondly, mental health burdens such as anxiety and distress have been found to be more prevalent in cancer survivors compared to the general population [65]. This can be attributed to the fear of potential recurrence and concerns about family and finances amongst other sources of distress [66] that cancer patients and survivors face. Such psychological stress has been linked to activation of the hypothalamic–pituitary–adrenal (HPA) axis and subsequently elevated glucocorticoid secretion [67]. Elevated cortisol concentrations have been associated with early miscarriage [68], due to direct adverse effects on the uterus and fetus, as well as the inhibition of hormones such as the pituitary luteinising hormone and progesterone secretion [64,69], both of which are important hormones in pregnancy. Progesterone plays an essential role in decidualization, the control of uterine contractions, as well as the regulation of maternal immune tolerance of the fetal semi-allograft, with reduced levels of progesterone in the luteal phase of menstruation and early gestation being linked to recurrent pregnancy loss.

Subgroup analyses revealed that cancer type is correlated with risks of preeclampsia, gestational diabetes and miscarriage. However, our study was unable to pinpoint the relationship between any specific cancers and the above obstetric outcomes, as most included studies investigated multiple cancers collectively. Future studies and treatment regimens could focus on individual cancer types to better delineate their specific impact on maternal health outcomes, providing clearer guidance for clinical management and patient counselling. Such stratification would also allow researchers to identify whether certain treatment modalities disproportionately affect reproductive function, resulting in poorer obstetric outcomes. For example, pelvic radiotherapy can lead to reduced uterine volume, impaired uterine distensibility due to myometrial fibrosis, uterine vasculature damage, and endometrial injury. Some factors that influence the degree of uterine damage include the total radiation dose, the site of irradiation, patient age at time of treatment, and whether a patient is pre- or post-pubertal [10], all of which may interact to determine subsequent obstetric risk. Understanding these nuances may support the development of tailored treatment and surveillance strategies for women planning for pregnancy after cancer, thereby enhancing survivorship care.

### 4.4. Limitations

Our study should be interpreted in due consideration of its limitations. Firstly, we anticipated high heterogeneity in the outcomes, which varied across different regions, with patients from a wide range of economic and sociocultural backgrounds included. There was also wide variation in cancer types included across the studies, further contributing to this heterogeneity. Secondly, treatment dosage and type were not consistent across studies, even within the same treatment modality. Thus, we were unable to quantify the true dose-dependent effect of each treatment modality on obstetric complications in this study. However, we were able to systematically review those studies. Future large-scale studies should investigate the effects of treatment modalities on this group of patients. Thirdly, we were unable to obtain individual patient data for this meta-analysis. A small number of studies also included populations consisting of in vitro fertilization and natural conceptions, which may affect outcomes. As such, we were also unable to obtain the staging of the cancers, which could potentially be a further contributing factor to these obstetric outcomes. Data regarding repeated pregnancies and other potential risk factors were also not available across studies. However, we were able to analyze the mediating effect of numerous key characteristics in the subgroups that we planned for. Lastly, our study focused exclusively on female cancer survivors to assess the impact of cancer and its treatments on maternal outcomes, particularly preeclampsia, gestational diabetes, and miscarriage. While we recognize the role of paternal factors, such as sperm quality, in miscarriage risk, and acknowledge other potential maternal factors that can impact subsequent pregnancy outcomes, such as pre-existing reproductive issues before cancer treatments, this data was not available in the literature. Future research on obstetric and maternal complications may also study paternal factors and examine outcomes of different conception methods, such as in vitro fertilization or natural conception, to provide a more comprehensive understanding of the impacts of cancer and gender on maternal outcomes in pregnancy.

## 5. Conclusions

In summary, this systematic review and meta-analysis demonstrates that female cancer survivors face an increased risk of preeclampsia, gestational diabetes, and miscarriage, likely mediated by persistent immunologic, vascular, metabolic, and psychosocial alterations induced by cancer and its treatment, with effects that may persist post-oncologic remission. Taken together, these findings further emphasize the need for targeted preconception counselling, vigilant individualized antenatal monitoring, and interdisciplinary survivorship care integrating oncology, obstetrics, and reproductive medicine to proactively identify and mitigate pregnancy-related risks. Early referral to specialized high-risk obstetricians may also be warranted for these cancer survivors. Further research delineating cancer- and treatment-specific risks will be essential to refine risk stratification, optimize reproductive outcomes, and advance the paradigm of holistic, life-course cancer survivorship.

## Figures and Tables

**Table 1:** Summary table for characteristics of included studies [13,14,15,26,27,28,29,30,31,32,33,34,35,36,37,38].

Author	Year of Publication	Region	Number of Cancer Survivors	Number of Controls	Control Characteristics	Type of Cancer	Treatment Modality	Mean Age of Diagnosis	Mean Age of Delivery	Mean Age of Treatment	Outcomes Studied
Cao	2022	Asia	96	192	Matched healthy	Thyroid	NR	NR	32.00	NR	Preeclampsia, gestational diabetes, anemia in pregnancy
Kao	2023	Asia	5151	51,503	Matched healthy	Mixed	Chemotherapy—29.99%	NR	32.82	NR	Preeclampsia, gestational diabetes, anemia in pregnancy
Nishikawa	2023	Asia	1102	98,714	Population	Mixed	NR	NR	31.01	NR	Hypertension in pregnancy
Rossi	2022	Europe	272	238	Matched healthy	ALL, LBL	NR	10.53	NR	NR	Miscarriage
Winther	2008	Europe	1479	5092	Siblings	Mixed	Radiation—33%	NR	NR	NR	Miscarriage
Madanat	2010	Europe	1309	5916	Siblings	Mixed	Chemotherapy—11.8% Radiation—33.16%	20.56	27.94	NR	Preeclampsia, gestational diabetes, hypertension in pregnancy
Vandijk	2020	Europe	786	853	Matched healthy	Mixed	Chemotherapy—50.1% Radiation—40.6%	6.40 (9.19)	28.50	NR	Miscarriage
Stensheim	2013	Europe	1196	90,632	Matched healthy	Mixed	NR	24.00 (5.10)	29.10 (4.90)	NR	Preeclampsia
Armuand	2019	Europe	278	829	Matched healthy	Breast	NR	11.40 (6.20)	27.60 (4.90)	NR	Preeclampsia, gestational diabetes, anemia in pregnancy, hypertension in pregnancy
Cao	2023	Europe	207	1019	Siblings	Colorectal	NR	28.30 (6.30)	33.70 (4.60)	NR	Preeclampsia, gestational diabetes
Iskender	2022	Europe	18	180	Matched healthy	Hematological	NR	NR	28.70 (5.70)	24.6	Preeclampsia, miscarriage
Mueller	2009	North America	1898	14,278	Matched healthy	Genital tract carcinoma	Chemotherapy—0.3% Radiation—0.3% Surgery—78.6%	16.06	23.24	NR	Preeclampsia, gestational diabetes, anemia in pregnancy
Niu	2019	North America	707	139,765	Population	Mixed	NR	NR	30.14 (6.13)	NR	Preeclampsia, gestational diabetes, anemia in pregnancy
Rubens	2022	North America	64,506	18,687,217	Population	Mixed	NR	NR	30.50	NR	Preeclampsia, gestational diabetes, hypertension in pregnancy, thromboembolism, maternal death
Anand	2022	North America	1024	2,468,625	Population and matched healthy	Leukemia and lymphoma	Chemotherapy—77.9% Radiation—53.2%	19.65	28.00	NR	Preeclampsia, hypertension in pregnancy
Haggar	2014	Oceania	1894	4138	Matched healthy	Mixed	Chemotherapy—20% Radiation—18% Surgery—34%	21.18	28.46	NR	Preeclampsia, gestational diabetes, anemia in pregnancy

## Data Availability

The data underlying this article are available in the article and in its online Supplementary Material.

## Supplementary Materials

The following supporting information can be downloaded at: https://www.mdpi.com/article/10.3390/cancers17243924/s1. Figure S1: Forest plot for risk of developing anemia in pregnancy among young cancer survivors; Figure S2: Forest plot for risk of developing hypertensive disorders of pregnancy among young cancer survivors; Figure S3: Forest plot for risk of developing miscarriages among young cancer survivors; Figure S4: Funnel plot for visual inspection of publication bias in studies assessing risk of preeclampsia among young cancer survivors; Figure S5: Trim-and-fill analysis for publication bias in studies assessing risk of preeclampsia among young cancer survivors; Figure S6: Quantitative assessment publication bias in studies assessing risk of preeclampsia among young cancer survivors; Figure S7: Funnel plot for visual inspection of publication bias in studies assessing risk of gestational diabetes among young cancer survivors; Figure S8: Trim-and-fill analysis for publication bias in studies assessing risk of gestational diabetes among young cancer survivors; Figure S9: Quantitative assessment publication bias in studies assessing risk of gestational diabetes among young cancer survivors; Figure S10: Leave-one-out analysis of studies assessing risk of preeclampsia among young cancer survivors, using the random effects model; Figure S11: Outlier assessment of studies assessing risk of preeclampsia among young cancer survivors, using the random effects model; Figure S12: Leave-one-out analysis of studies assessing risk of gestational diabetes among young cancer survivors, using the random effects model; Figure S13: Outlier assessment of studies assessing risk of gestational diabetes among young cancer survivors, using the random effects model. Table S1: Search strategy; Table S2: Meta-analyses of young cancer survivors on risk of developing preeclampsia stratified by categorical study-level characteristics using the random effect model; Table S3: Meta-analyses of young cancer survivors on risk of developing gestational diabetes stratified by categorical study-level characteristics using the random effect model; Table S4: Mixed effects meta-regression of risk ratio against potential effect moderators (categorical study-level characteristics) for risk of developing preeclampsia and gestational diabetes in young cancer survivors; Table S5: Evaluation of the mediating or confounding effect of treatment modality on maternal outcomes, including preeclampsia, gestational diabetes, anemia, hypertension in pregnancy, and miscarriage; Table S6: Evaluation of the mediating or confounding effect of type of cancer on maternal outcomes, including preeclampsia, gestational diabetes, anemia, hypertensive disorders of pregnancy, and miscarriage; Table S7: Quality assessment of included cohort studies using the Joanna Brigg’s Institute Critical Appraisal tool; Table S8: Certainty of evidence assessed using the GRADE framework.
