# Supplementary material for: Obstetric Complications and Pregnancy Outcomes in Cancer Survivors: A Systematic Review and Meta-Analysis
Source: Cancers (Basel). 2025 Dec 8;17(24):3924. doi: 10.3390/cancers17243924 (PMC12731054; doi:10.3390/cancers17243924)

**Supplementary Material**

Supplementary Table S1: Search strategy
Supplementary Table S2: Meta-analyses of young cancer survivors on risk of developing preeclampsia stratified by categorical study-level characteristics using the random effect model
Supplementary Table S3: Meta-analyses of young cancer survivors on risk of developing gestational diabetes stratified by categorical study-level characteristics using the random effect model
Supplementary Table S4: Mixed effects meta-regression of risk ratio against potential effect moderators (categorical study-level characteristics) for risk of developing preeclampsia and gestational diabetes in young cancer survivors
Supplementary Table S5: Evaluation of the mediating or confounding effect of treatment modality on maternal outcomes, including preeclampsia, gestational diabetes, anemia, hypertension in pregnancy, and miscarriage
Supplementary Table S6: Evaluation of the mediating or confounding effect of type of cancer on maternal outcomes, including preeclampsia, gestational diabetes, anemia, hypertensive disorders of pregnancy, and miscarriage
Supplementary Table S7: Quality assessment of included cohort studies using the Joanna Brigg's Institute Critical Appraisal tool
Supplementary Table S8: Certainty of evidence assessed using the GRADE framework

Supplementary Figure S1: Forest plot for risk of developing anemia in pregnancy among young cancer survivors
Supplementary Figure S2: Forest plot for risk of developing hypertensive disorders of pregnancy among young cancer survivors
Supplementary Figure S3: Forest plot for risk of developing miscarriages among young cancer survivors
Supplementary Figure S4: Funnel plot for visual inspection of publication bias in studies assessing risk of preeclampsia among young cancer survivors
Supplementary Figure S5: Trim-and-fill analysis for publication bias in studies assessing risk of preeclampsia among young cancer survivors
Supplementary Figure S6: Quantitative assessment publication bias in studies assessing risk of preeclampsia among young cancer survivors
Supplementary Figure S7: Funnel plot for visual inspection of publication bias in studies assessing risk of gestational diabetes among young cancer survivors
Supplementary Figure S8: Trim-and-fill analysis for publication bias in studies assessing risk of gestational diabetes among young cancer survivors
Supplementary Figure S9: Quantitative assessment publication bias in studies assessing risk of gestational diabetes among young cancer survivors
Supplementary Figure S10: Leave-one-out analysis of studies assessing risk of preeclampsia among young cancer survivors, using the random effects model
Supplementary Figure S11: Outlier assessment of studies assessing risk of preeclampsia among young cancer survivors, using the random effects model
Supplementary Figure S12: Leave-one-out analysis of studies assessing risk of gestational diabetes among young cancer survivors, using the random effects model
Supplementary Figure S13: Outlier assessment of studies assessing risk of gestational diabetes among young cancer survivors, using the random effects model

Supplementary Table S1: Search strategy

| Database | Search terms | Results |
|---|---|---|
| PubMed | ('pregnancy outcome*'[tiab] OR 'reproduc*'[tiab] OR 'abort*'[tiab] OR 'miscarriage'[tiab] OR 'preeclampsia'[tiab] OR ‘anemia’[tiab] OR ‘anaemia’[tiab] OR ‘diabetes’[tiab] OR ‘mellitus’[tiab])<br>AND<br>(‘obstetric*’[tiab] OR ‘maternal’[tiab] OR ‘maternity’[tiab] OR ‘mother*’[tiab] OR ‘parent*’[tiab] OR ‘gestation*’[tiab] OR ‘pregnan*’[tiab])<br>AND<br>('neoplas*'[tiab] OR 'neoplasms'[MeSH Terms] OR 'cancer'[tiab] OR 'tumo*'[tiab] OR 'malignan*'[tiab] OR ‘leukemia[tiab])<br>AND<br>('child'[tiab] OR 'adolescent'[tiab] OR 'infant'[tiab] OR 'young adult'[tiab] OR 'survivor*'[tiab] OR 'childhood cancer survivor'[tiab]) | 1,651 |
| Embase | ('pregnancy outcome*':ab,ti OR 'reproduc*':ab,ti OR 'abort*':ab,ti OR ‘anemia’:ab,ti OR ‘anaemia’:ab,ti OR ‘diabetes’:ab,ti OR ‘mellitus’:ab,ti OR 'miscarriage':ab,ti OR 'preeclampsia':ab,ti)<br>AND<br>(‘obstetric*’:ab,ti OR ‘maternal’:ab,ti OR ‘maternity’:ab,ti OR ‘mother*’:ab,ti OR ‘parent*’:ab,ti OR ‘gestation*’:ab,ti OR ‘pregnan*’:ab,ti)<br>AND<br>('neoplas*':ab,ti OR 'neoplasm':ab,ti OR 'cancer':ab,ti OR 'tumo*':ab,ti OR 'malignan*':ab,ti OR 'leukemia':ab,ti)<br>AND<br>('child':ab,ti OR 'adolescent':ab,ti OR 'infant':ab,ti OR 'young adult':ab,ti OR 'survivor*':ab,ti OR 'childhood cancer survivor':ab,ti)<br>AND<br>[2000-2024]/py | 2,879 |

Supplementary Table S2: Meta-analyses of young cancer survivors on risk of developing preeclampsia stratified by categorical study-level characteristics using the random effect model

| Variable | Cohorts | $N_1$ Intervention | $N_2$ Controls | Risk Ratio | 95% CI | I2 | Test of interaction (p-value) |
|---|---|---|---|---|---|---|---|
| Overall | 11 | 76,260 | 18,995,669 | 1.37 | 1.17; 1.62 | 48% | NA |
| Control = Matched | 7 | 10,531 | 161,752 | 1.34 | 1.11; 1.62 | 50% | 0.20 |
| Control = Siblings | 2 | 1,516 | 6,935 | 2.15 | 1.24; 3.71 | 0% | |
| Control = Population | 2 | 65,213 | 18,826,982 | 1.24 | 0.96; 1.60 | 35% | |
| Region = Asia | 2 | 5,247 | 51,695 | 1.17 | 0.88; 1.56 | 31% | 0.14 |
| Region = Europe | 5 | 3,008 | 98,576 | 1.75 | 1.34; 2.28 | 43% | |
| Region = North America | 3 | 67,111 | 18,841,260 | 1.23 | 1.02; 1.48 | 12% | |
| Region = Oceania | 1 | 1,894 | 4,138 | 1.36 | 1.01; 1.83 | NA | |
| Type of Cancer = Thyroid | 1 | 96 | 192 | 2.50 | 0.69; 9.10 | NA | **0.04** |
| Type of Cancer = Various | 6 | 75,465 | 18,902,817 | 1.26 | 1.17; 1.35 | 36% | |
| Type of Cancer = NR | 1 | 1,196 | 90,632 | 1.37 | 1.08; 1.74 | NA | |
| Type of Cancer = Breast | 1 | 278 | 829 | 3.17 | 1.62; 6.19 | NA | |
| Type of Cancer | 1 | 207 | 1,019 | 2.24 | 1.24; 4.04 | NA | |

| | | | | | | | |
|---|---|---|---|---|---|---|---|
| = Colorectal | | | | | | | |
| Type of Cancer = Hematological | 1 | 18 | 180 | 1.82 | 0.44; 7.57 | NA | |
| Age of diagnosis NR | 5 | 70,478 | 18,878,857 | 1.23 | 0.97; 1.57 | 46% | 0.44 |
| Age of diagnosis <21 | 4 | 5,379 | 25,161 | 1.51 | 1.12; 2.03 | 63% | |
| Age of diagnosis >21 | 2 | 1,403 | 91,651 | 1.59 | 1.07; 2.35 | 55% | |
| Age of delivery >30 | 5 | 70,667 | 18,879,696 | 1.30 | 1.03; 1.65 | 62% | 0.48 |
| Age of delivery <30 | 6 | 6,593 | 115,973 | 1.48 | 1.15; 1.90 | 39% | |
| Smoke = NR | 5 | 3,911 | 234,907 | 1.34 | 0.99; 1.81 | 0% | 0.82 |
| Smoke = >10% | 2 | 3,207 | 20,194 | 1.32 | 0.82; 2.13 | 17% | |
| Smoke = <10% | 4 | 70,142 | 18,740,568 | 1.51 | 1.13; 2.02 | 80% | |

Supplementary Table S3: Meta-analyses of young cancer survivors on risk of developing gestational diabetes stratified by categorical study-level characteristics using the random effect model

| **Variable** | **Cohorts** | **$N_1$ Intervention** | **$N_2$ Controls** | **Risk Ratio** | **95% CI** | **I2** | **Test of interaction (p-value)** |
|---|---|---|---|---|---|---|---|
| Overall | 9 | 76,046 | 18,904,857 | 1.29 | 1.05; 1.59 | 80% | NA |
| Control = Matched | 5 | 9,317 | 70,940 | 1.35 | 0.98; 1.86 | 89% | 0.94 |
| Control = Sibling | 2 | 1,516 | 6,935 | 1.24 | 0.68; 2.27 | 0% | |
| Control = Population | 2 | 65,213 | 18,826,982 | 1.23 | 0.80; 1.89 | 0% | |
| Region = Asia | 2 | 5,247 | 51,695 | 1.05 | 0.96; 1.16 | 0% | **<0.01** |
| Region = Europe | 3 | 1,794 | 7,764 | 1.21 | 0.84; 1.74 | 0% | |
| Region = North America | 3 | 67,111 | 18,841,260 | 1.25 | 1.21;1.28 | 0% | |
| Region = Oceania | 1 | 1,894 | 4,138 | 2.66 | 2.00; 3.54 | NA | |
| Type of Cancer = Thyroid | 1 | 96 | 192 | 1.08 | 0.67; 1.72 | NA | 0.96 |
| Type of Cancer = Various | 6 | 75,465 | 18,902,817 | 1.31 | 0.99; 1.74 | 87% | |
| Type of Cancer = Breast | 1 | 278 | 829 | 1.33 | 0.41; 4.27 | NA | |
| Type of Cancer = Colorectal | 1 | 207 | 1,019 | 1.54 | 0.57; 4.15 | NA | |

| | | | | | | | |
|---|---|---|---|---|---|---|---|
| Age of diagnosis = NR | 4 | 70,460 | 18,878,677 | 1.15 | 0.87; 1.52 | 73% | 0.44 |
| Age of diagnosis < 21 | 4 | 5,379 | 25,161 | 1.53 | 1.08; 2.16 | 84% | |
| Age of diagnosis >21 | 1 | 207 | 1,019 | 1.54 | 0.57; 4.15 | NA | |
| Age of delivery = >30 | 5 | 70,667 | 18,879,696 | 1.17 | 0.91; 1.50 | 64% | 0.19 |
| Age of delivery = <30 | 4 | 5,379 | 25,161 | 1.54 | 1.11; 2.14 | 84% | |
| Smoke = NR | 3 | 2,697 | 144,095 | 1.57 | 1.12; 2.20 | 89% | 0.35 |
| Smoke = >10% | 2 | 3,207 | 20,194 | 1.09 | 0.69; 1.71 | 0% | |
| Smoke = <10% | 4 | 70,142 | 18,740,568 | 1.19 | 0.86; 1.63 | 72% | |

Supplementary Table S4: Mixed effects meta-regression of risk ratio against potential effect moderators (categorical study-level characteristics) for risk of developing preeclampsia and gestational diabetes in young cancer survivors

| | **Ratio** | **P** | **95% CI Lower** | **95% CI Upper** | **$I^2$ (% residual heterogeneity)** |
|---|---|---|---|---|---|
| **Preeclampsia** | | | | | |
| Age of diagnosis <21 | 0.2514 | 0.8297 | -0.5469 | 0.4388 | 75.43% |
| Age of delivery >30 | 0.1762 | 0.4752 | -0.4713 | 0.2196 | 75.83% |
| Smoke >10% | 0.2846 | 0.6467 | -0.6884 | 0.4274 | 80.25% |
| **Gestational Diabetes** | | | | | |
| Age of diagnosis <21 | 0.5943 | 0.9899 | -1.1723 | 1.1573 | 91.03% |
| Age of delivery >30 | 0.2111 | 0.1946 | -0.6875 | 0.1399 | 88.06% |
| Smoke >10% | 0.2820 | 0.7603 | -0.6387 | 0.4667 | 89.50% |

Supplementary Table S5: Evaluation of the mediating or confounding effect of treatment modality on maternal outcomes, including preeclampsia, gestational diabetes, anemia, hypertension in pregnancy, and miscarriage

| Author | Year | Country | Study population | Key findings |
|---|---|---|---|---|
| **Preeclampsia** | | | | |
| **Cao** | **2022** | **China** | 96 women who are thyroid cancer survivors and 192 women from controls, mean age of delivery of 32, recruited from West China Second University Hospital of Sichuan University. | No significantly risk for developing preeclampsia for patients treated with radiotherapy (OR: 6.50, 95%CI: 0.65-65.10). |
| **Kao** | **2023** | **Taiwan** | 5,151 births from young cancer survivors and 51,503 births from controls, mean age of delivery of 32.82, recruited from Taiwan National Health Insurance (NHI) and Taiwan Birth Reporting System (TBRS). | No significant risk for developing preeclampsia for patients treated with radiotherapy (OR: 0.79, 95% CI: 0.40-1.47) or chemotherapy with/without radiotherapy (OR: 1.21, 95%CI: 0.96-1.54). |
| **Iskender** | **2022** | **Turkey** | 18 young cancer survivors and 180 controls, mean age of delivery of 28.70(5.70), recruited from Abdurrahman Yurtarslan Oncology Hospital database. | No significant risk for developing preeclampsia in patients (14.3%), compared to controls (7.2%), after undergoing stem cell transplantation (p = 0.343). |
| **Gestational Diabetes** | | | | |
| **Cao** | **2022** | **China** | 96 women who are thyroid cancer survivors and 192 women from controls, mean age of delivery of 32, recruited from West China Second University Hospital of Sichuan University. | No significantly risk for developing gestational diabetes for patients treated with radiotherapy (OR: 0.83, 95%CI: 0.28-2.39). |
| **Kao** | **2023** | **Taiwan** | 5,151 births from young cancer survivors and 51,503 births from controls, mean age of delivery of 32.82, | No significant risk for developing gestational diabetes for patients treated with radiotherapy (OR: 0.85, 95%CI: 0.55- |

| | | | | |
|---|---|---|---|---|
| | | | recruited from Taiwan National Health Insurance (NHI) and Taiwan Birth Reporting System (TBRS). | 1.31) or chemotherapy with/without radiotherapy (OR: 0.94, 95%CI: 0.77-1.14). |
| **Mueller** | **2009** | **USA** | 1,898 young cancer survivors and 14,278 live births from controls, mean age of diagnosis 16.06, mean age of delivery 23.24, recruited from Surveillance, Epidemiology, and End Results(SEER) Program of the National Cancer Institute, comprising of populations from the Cancer Surveillance System of Western Washington in Seattle; the Karmanos Cancer Institute of Wayne State University in Detroit, Michigan; the Utah Cancer Registry at the University of Utah in Salt Lake City; and the SEER registry in Atlanta, Georgia. | No significant risk of developing gestational diabetes for patients treated with radiotherapy (OR: 0.90, 95%CI: 0.29-2.81) and chemotherapy (OR: 1.26, 95%CI: 0.53-3.04). |
| **Haggar** | **2014** | **Australia** | 1,894 births and 4,138 controls, mean age of diagnosis 21.18, mean age of delivery 28.46 recruited from The Western Australian Data Linkage System (WADLS). | Significantly increased risk for developing gestational diabetes for patients treated with chemoradiation (OR: 2.52, 95%CI: 1.12-5.09) but no significant risk for those treated with radiotherapy alone (OR: 0.80. 95%CI: 0.25-2.56) or chemotherapy alone (OR: 1.25, 95%CI: 0.31-4.99). |
| **Anemia in pregnancy** | | | | |
| **Cao** | **2022** | **China** | 96 women who are thyroid cancer survivors and 192 women from controls, mean age of delivery of 32 recruited from West China Second University Hospital of Sichuan University. | No significant risk for developing anemia in pregnancy for patients treated with radiotherapy (OR: 1.29, 95%CI: 0.39, 4.32). |
| **Kao** | **2023** | **Taiwan** | 5,151 births from young cancer survivors and 51,503 births from controls, mean age of delivery of 32.82, recruited from Taiwan National Health Insurance (NHI) and Taiwan Birth Reporting System (TBRS). | No significant risk for developing anemia in pregnancy for patients treated with radiotherapy (OR: 1.44, 95%CI: 0.92-2.23) or chemotherapy with/without radiotherapy (OR: 1.01, 95%CI: 0.80-2.28). |

| | | | | |
|---|---|---|---|---|
| **Mueller** | **2009** | **USA** | 1,898 young cancer survivors and 14,278 live births from controls, mean age of diagnosis 16.06, mean age of delivery 23.24, recruited from Surveillance, Epidemiology, and End Results(SEER) Program of the National Cancer Institute, comprising of populations from the Cancer Surveillance System of Western Washington in Seattle; the Karmanos Cancer Institute of Wayne State University in Detroit, Michigan; the Utah Cancer Registry at the University of Utah in Salt Lake City; and the SEER registry in Atlanta, Georgia. | No significant risk for developing anemia in pregnancy for patients treated with radiotherapy (OR: 0.97, 95%CI: 0.40-2.32) and chemotherapy (OR: 1.39, 95%CI: 0.73-2.69). |
| **Hypertension in Pregnancy** | | | | |
| **Anand** | **2022** | **USA** | 1,539 young cancer survivors and 2,469,639 controls, mean age of diagnosis of 19.65, mean age of delivery of 28.00, recruited from the California Office of Statewide Health Planning and Development (OSHPD) and Surveillance, Epidemiology, and End Results (SEER) cancer registry data in Iowa. | In the Iowa cohort, there was no significant risk of developing hypertension in pregnancy for patients treated with radiation only (OR: 1.98, 95%CI: 0.63-6.22) or chemotherapy with radiation (OR: 1.33, 95%CI: 0.57-3.15), when compared to those who did chemotherapy alone. |
| **Miscarriage** | | | | |
| **Winther** | **2008** | **Denmark** | 1,479 young cancer survivors and 5,092 sibling controls, recruited from Danish Cancer Registry. | Significantly increased risk of developing miscarriages for young cancer survivors who were treated with radiotherapy (OR: 1.58, 95%CI: 1.15-2.17), especially those who received irradiation to the ovaries and uterus (PR:2.8, 95% CI, 1.7-4.7) and pituitary gland (PR:1.8, 95%CI: 1.1-3.0). |
| **Vandijk** | **2020** | **Netherlands** | 592 live births from young cancer survivors and 651 births from controls, mean age at diagnosis of 6.40(9.19) mean age at delivery of 28.5, recruited from DCOG-LATER-VEVO study. | No significant risk for developing miscarriage for patients treated with cranial/spinal radiotherapy (OR: 1.30, 95%CI: 0.80-2.20), lower abdominal pelvic radiotherapy (OR: 1.80, 95%CI: 0.90-3.50) and chemotherapy, CED score of |

| | | | | |
|---|---|---|---|---|
| | | | | 0-4000mg/$m^2$ (OR: 1.40 ,95%CI: 0.80-2.50) and >8000mg/m2 (OR: 0.80, 95%CI: 0.50-1.40). |
| **Iskender** | **2022** | **Turkey** | 18 young cancer survivors and 180 controls, mean age of delivery of 28.7(5.7), recruited from Abdurrahman Yurtarslan Oncology Hospital database. | No significant risk of developing miscarriage for young cancer survivors (22.2%) compared to controls (15%) after undergoing stem cell transplantation (p=0.421). |

Supplementary Table S6: Evaluation of the mediating or confounding effect of type of cancer on maternal outcomes, including preeclampsia, gestational diabetes, anemia, hypertensive disorders of pregnancy, and miscarriage

| **Author** | **Year** | **Country** | **Study population** | **Key findings** |
|---|---|---|---|---|
| | | **Preeclampsia** | | |
| **Cao** | **2022** | **China** | 96 women who are thyroid cancer survivors and 192 women from controls, mean age at delivery of 32, recruited from West China Second University Hospital of Sichuan University. | No significant risk of developing preeclampsia among thyroid cancer survivors (OR: 2.779, 95%CI: 0.708-10.91). |
| **Armuand** | **2019** | **Sweden** | 278 young cancer survivors and 829 controls, mean age of diagnosis 11.4(6.2), mean age of delivery 27.6(4.9), were recruited from the Swedish Cancer Registry. | Significantly increased risk for developing preeclampsia for those with hematological cancers (OR: 4.66, 95%CI:1.34 to 16.21) and neuroendocrine tumors (OR 13.02, 95%CI:1.13 to 150.53). |
| **Cao** | **2023** | **Sweden** | 207 cancer survivors and 1,019 controls, mean age of diagnosis 28.3(6.3), mean age of delivery 33.7(4.6) were recruited from the Swedish Cancer Registry. | Significantly increased risk of developing preeclampsia for survivors of colorectal cancer. (OR: 2.40, 95%CI: 1.27-4.55). |

| | | | | |
|---|---|---|---|---|
| **Mueller** | **2009** | **USA** | 1,898 young cancer survivors and 14,278 live births from controls, mean age of diagnosis 16.06, mean age of delivery 23.24, recruited from Surveillance, Epidemiology, and End Results(SEER) Program of the National Cancer Institute, comprising of populations from the Cancer Surveillance System of Western Washington in Seattle; the Karmanos Cancer Institute of Wayne State University in Detroit, Michigan; the Utah Cancer Registry at the University of Utah in Salt Lake City; and the SEER registry in Atlanta, Georgia. | No significant risk of developing preeclampsia for survivors of genital tract carcinomas (OR: 0.99, 95%CI: 0.70-1.41) or other cancers. |
| | | **Gestational Diabetes** | | |
| **Cao** | **2022** | **China** | 96 women who are thyroid cancer survivors and 192 women from controls, mean age at delivery of 32, recruited from West China Second University Hospital of Sichuan University. | No significant risk of developing gestational diabetes among thyroid cancer survivors (OR: 1.126, 95%CI: 0.609-2.084). |
| **Cao** | **2023** | **Sweden** | 207 cancer survivors and 1,019 controls, mean age of diagnosis 28.3(6.3), mean age of delivery 33.7(4.6) were recruited from the Swedish Cancer Registry. | No significant risk of developing gestational diabetes for survivors of colorectal cancer (OR: 1.53, 95%CI: 0.55-4.25). |
| **Mueller** | **2009** | **USA** | 1,898 young cancer survivors and 14,278 live births from controls, mean age of diagnosis 16.06, mean age of delivery 23.24, recruited from Surveillance, Epidemiology, and End Results(SEER) Program of the National Cancer Institute, comprising of populations from the Cancer Surveillance System of Western Washington in Seattle; the Karmanos Cancer Institute of Wayne State University in Detroit, Michigan; the Utah Cancer Registry at the University of Utah in Salt Lake City; and the SEER registry in Atlanta, Georgia. | Significantly increased risk of developing gestational diabetes for survivors of bone tumors (OR: 4.92, 95%CI: 1.60-15.13), but no significant risk for survivors of genital tract carcinomas (OR: 0.86, 95%CI: 0.49-1.53), leukemia (OR: 1.47, 95%CI: 0.37-5.83), lymphoma (OR: 0.41, 95%CI: 0.06-2.95), central nervous system tumors (OR: 0.85, 95%CI: 0.48-1.49), embryonal cancers (OR: 0.65, 95%CI: 0.28-1.47), soft tissue sarcoma (OR: 1.44, 95%CI: 0.89-2.33), germ cell tumors (OR: 1.36, 95%CI: 0.81-2.29), thyroid carcinoma (OR: 1.11, 95%CI: 0.72-1.73), non-basal/squamous cell skin cancers (OR: 1.39, , 95%CI: 0.90-2.15) and other carcinomas (OR: 1.47, 95%CI: 0.85-2.55). |

| | | | | |
|---|---|---|---|---|
| **Haggar** | **2014** | **Australia** | 1,894 births and 4,138 controls, mean age of diagnosis 21.18, mean age of delivery 28.46 recruited from The Western Australian Data Linkage System (WADLS). | Significantly increased risk of developing gestational diabetes for young survivors of central nervous system tumors (OR: 2.32, 95%CI: 1.29-3.98), bone cancer (OR: 2.14, 95%CI: 1.12-6.08), carcinomas (OR: 2.45, 95%CI: 1.28-4.04) but no significant risk for young survivors of leukemia (OR: 1.23, 95%CI: 0.19-9.44), lymphoma (OR: 1.52, 95%CI: 0.78-2.83), soft tissue sarcoma (OR: 2.73, 95%CI: 0.41-18.8), germ cell tumors (OR: 2.91, 95%CI: 0.52-18.8) and melanoma (OR: 2.01, 95%CI: 0.34-12.8). |
| **Anemia in Pregnancy** | | | | |
| **Cao** | **2022** | **China** | 96 women who are thyroid cancer survivors and 192 women from controls, mean age at delivery of 32, recruited from West China Second University Hospital of Sichuan University. | No significant risk of developing anemia during pregnancy among thyroid cancer survivors (OR: 0.668, 95%CI: 0.292-1.529). |
| **Mueller** | **2009** | **USA** | 1,898 young cancer survivors and 14,278 live births from controls, mean age of diagnosis 16.06, mean age of delivery 23.24, recruited from Surveillance, Epidemiology, and End Results(SEER) Program of the National Cancer Institute, comprising of populations from the Cancer Surveillance System of Western Washington in Seattle; the Karmanos Cancer Institute of Wayne State University in Detroit, Michigan; the Utah Cancer Registry at the University of Utah in Salt Lake City; and the SEER registry in Atlanta, Georgia. | Significantly increased risk of developing anemia in pregnancy among survivors of central nervous system tumors (OR: 3.05, 95%CI: 1.16-7.98) but no significant risk for survivors of leukemia (OR: 2.14, 95%CI: 0.82-5.00), lymphoma (OR: 1.00, 95%CI: 0.34-2.97), embryonal cancer (OR: 1.55, 95%CI: 0.36-6.68), bone cancers (OR: 0.92, 95%CI: 0.14-6.08), thyroid cancer (OR: 0.71, 95%CI: 0.10-4.93), non-basal/squamous cell cancer (OR: 1.22, 95%CI: 0.17-8.59) and other carcinomas (OR:1.13, 95%CI: 0.17-7.68). |
| **Hypertension in Pregnancy** | | | | |
| **Nishikawa** | **2023** | **Japan** | 1,102 young cancer survivors and 98,714 controls, mean age of delivery of 31.01, recruited from the Japan Environment and Children's Study (JECS) cohort. | No significant risk for survivors of cervical cancer to developing mild hypertensive disorders of pregnancy (OR: 0.75, 95%CI: 0.45-1.26) or severe hypertensive disorders of pregnancy (OR: 0.96, 95%CI: 0.47-1.93). |

| Miscarriage | | | | |
|---|---|---|---|---|
| **Cao** | **2022** | **China** | 96 women who are thyroid cancer survivors and 192 women from controls, mean age at delivery of 32, recruited from West China Second University Hospital of Sichuan University. | No significant risk of developing late miscarriage among thyroid cancer survivors (OR: 2.011, 95%CI: 0.124-32.496). |
| **Winther** | **2008** | **Denmark** | 1,479 young cancer survivors and 5,092 sibling controls, recruited from Danish Cancer Registry. | Significantly increased risk for developing miscarriages for young cancer survivors of Wilms and other renal tumors (PR: 3.00, 95%CI:1.60-5.50), germ cell, trophoblastic and other gonadal neoplasm (PR: 2.70, 95%CI: 1.40-5.20), and no significant risk for survivors of leukemia (PR: 1.20, 95%CI: 0.70-2.00), lymphoma (OR: 1.20, 95%CI: 0.80-2.00), central nervous system tumors (PR: 1.30, 95%CI: 0.90-1.90), sympathetic nervous system tumors (PR: 1.70, 95%CI: 0.80-4.00), retinoblastoma (OR: 1.30, 95%CI: 0.70-2.40), bone cancers (PR: 0.60, 95%CI: 0.20-2.20), soft tissue sarcoma (PR: 1.10, 95%CI: 0.60-2.00), and carcinoma/other malignant epithelial cancers (PR: 0.9, 95%CI: 0.50-1.20). |
| **Vandijk** | **2020** | **Netherlands** | 592 live births from young cancer survivors and 651 births from controls, mean age at diagnosis of 6.40(9.19), mean age at delivery of 28.50, recruited from DCOG-LATER-VEVO study. | Significantly increased risk of developing miscarriages for young cancer survivors of leukemia (OR: 1.60, 95%CI: 1.10-2.30), but no significant risk for those with lymphoma (OR: 0.80, 95%CI: 0.40-1.30), central nervous system tumors (OR: 1.30, 95%CI: 0.50-3.40), neuroblastoma/other peripheral nerve tumors (OR: 0.70, 95%CI: 0.30-2.10), renal tumors (OR: 1.90, 95%CI: 1.00-3.50), bone cancers (OR: 0.50, 95%CI: 0.20-1.60), soft tissue sarcoma (OR: 1.20, 95%CI: 0.60-2.50) and germ cell tumors (OR: 1.50, 95%CI: 0.50-4.40). |

Supplementary Table S7: Quality assessment of included cohort studies using the Joanna Brigg's Institute Critical Appraisal tool

| Study | 1 | 2 | 3 | 4 | 5 | 6 | 7 | 8 | 9 | 10 | 11 |
|---|---|---|---|---|---|---|---|---|---|---|---|
| **Cao 2022** | NA | Y | Y | Y | Y | Y | Y | U | U | U | Y |
| **Kao 2023** | NA | Y | Y | Y | Y | Y | Y | Y | Y | NA | Y |
| **Nishikawa 2023** | NA | Y | Y | Y | Y | Y | Y | Y | Y | NA | Y |
| **Rossi 2022** | NA | Y | Y | Y | Y | Y | Y | Y | Y | NA | Y |
| **Winther 2008** | NA | Y | Y | Y | Y | Y | Y | Y | U | U | Y |
| **Madanat 2010** | NA | Y | Y | Y | Y | Y | Y | Y | Y | NA | Y |
| **Vandijk 2020** | NA | Y | Y | Y | Y | Y | Y | Y | Y | NA | Y |
| **Stensheim 2013** | NA | Y | Y | Y | Y | Y | Y | Y | Y | NA | Y |
| **Armuand 2019** | NA | Y | Y | Y | Y | Y | Y | Y | Y | NA | Y |
| **Cao 2023** | NA | Y | Y | Y | Y | Y | Y | Y | Y | NA | Y |
| **Iskender 2022** | NA | Y | Y | Y | Y | Y | Y | Y | Y | NA | Y |
| **Mueller 2009** | NA | Y | Y | Y | Y | Y | Y | Y | U | U | Y |

| | | | | | | | | | | | |
|---|---|---|---|---|---|---|---|---|---|---|---|
| **Niu 2019** | NA | Y | Y | Y | Y | Y | Y | Y | Y | NA | Y |
| **Rubens 2022** | NA | Y | Y | Y | Y | Y | Y | Y | Y | NA | Y |
| **Anand 2022** | NA | Y | Y | Y | Y | Y | Y | U | U | U | Y |
| **Haggar 2014** | NA | Y | Y | Y | Y | Y | Y | Y | Y | NA | Y |

| Checklist |
|---|
| 1. Were the two groups similar and recruited from the same population? |
| 2. Were the exposures measured similarly to assign people to both exposed and unexposed groups? |
| 3. Was the exposure measured in a valid and reliable way? |
| 4. Were confounding factors identified? |
| 5. Were strategies to deal with confounding factors stated? |
| 6. Were the groups/participants free of the outcome at the start of the study (or at the moment of exposure)? |
| 7. Were the outcomes measured in a valid and reliable way? |
| 8. Was the follow up time reported and sufficient to be long enough for outcomes to occur? |
| 9. Was follow up complete, and if not, were the reasons to loss to follow up described and explored? |
| 10. Were strategies to address incomplete follow up utilized? |
| 11. Was appropriate statistical analysis used? |

Legend:
Y – Yes
N – No
U – Unclear
NA – Not applicable

Supplementary Table S8: Certainty of evidence assessed using the GRADE framework

| Outcome | Study Design | Risk of Bias | Inconsistency | Indirectness | Imprecision | Other Considerations | Grade |
|---|---|---|---|---|---|---|---|
| Preeclampsia | Controlled observational | Not serious | Not serious | Not serious | Not serious | Small effect size | Moderate |
| Gestational diabetes | Controlled observational | Not serious | Not serious | Not serious | Not serious | Small effect size | Moderate |
| Miscarriage | Controlled observational | Not serious | Not serious | Not serious | Not serious | Small effect size | Moderate |
| Anemia | Controlled observational | Not serious | Not serious | Not serious | Not serious | No significant effect size | Moderate |
| Hypertension | Controlled observational | Not serious | Not serious | Not serious | Not serious | No significant effect size | Moderate |

Supplementary Figure S1: Forest plot for risk of developing anemia in pregnancy among young cancer survivors

| Study | Experimental Events | Experimental Total | Control Events | Control Total | RR | 95%-CI | Weight |
|---|---|---|---|---|---|---|---|
| Cao | 10 | 96 | 27 | 192 | 0.74 | [0.37; 1.47] | 5.7% |
| Armuand 2019 | 16 | 278 | 44 | 829 | 1.08 | [0.62; 1.89] | 8.0% |
| Kao 2023 | 289 | 5151 | 2345 | 51503 | 1.23 | [1.09; 1.39] | 34.5% |
| Niu | 103 | 707 | 14315 | 139765 | 1.42 | [1.19; 1.70] | 28.7% |
| Haggar | 21 | 1894 | 39 | 4138 | 1.18 | [0.69; 1.99] | 8.7% |
| Mueller | 30 | 1898 | 269 | 14278 | 0.84 | [0.58; 1.22] | 14.3% |
| **Random effects model** | | **10024** | | **210705** | **1.16** | **[0.98; 1.39]** | **100.0%** |

Heterogeneity: $I^2 = 44\%$, $\tau^2 = 0.0195$, $p = 0.11$

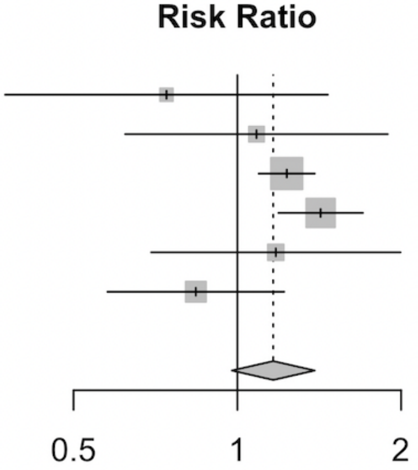


Supplementary Figure S2: Forest plot for risk of developing hypertensive disorders of pregnancy among young cancer survivors

| Study | Experimental Events | Experimental Total | Control Events | Control Total | RR | 95%-CI | Weight |
|---|---|---|---|---|---|---|---|
| Madanat 2010 | 88 | 1309 | 265 | 5916 | 1.50 | [1.19; 1.90] | 26.6% |
| Armuand 2019 | 5 | 278 | 7 | 829 | 2.13 | [0.68; 6.66] | 3.0% |
| Rubens | 2774 | 64506 | 784863 | 18687217 | 1.02 | [0.99; 1.06] | 39.8% |
| Anand | 107 | 1539 | 143690 | 2469639 | 1.19 | [1.00; 1.43] | 30.6% |
| **Random effects model** | | **67632** | | **21163601** | **1.21** | **[0.99; 1.49]** | **100.0%** |

Heterogeneity: $I^2 = 78\%$, $\tau^2 = 0.0274$, $p < 0.01$

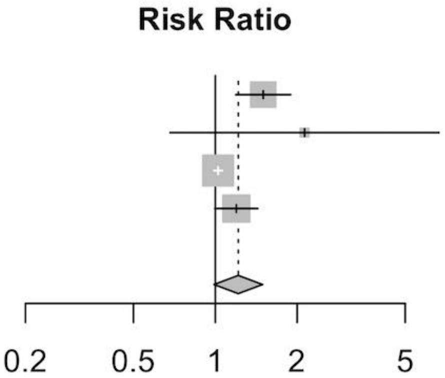

Supplementary Figure S3: Forest plot for risk of developing miscarriages among young cancer survivors

| Study | Experimental Events | Experimental Total | Control Events | Control Total | RR | 95%-CI | Weight |
|---|---|---|---|---|---|---|---|
| Rossi | 16 | 85 | 10 | 61 | 1.15 | [0.56; 2.36] | 4.1% |
| Vandijk | 138 | 786 | 138 | 853 | 1.09 | [0.88; 1.35] | 45.8% |
| Iskender | 4 | 18 | 27 | 180 | 1.48 | [0.58; 3.76] | 2.4% |
| Winther | 109 | 1473 | 304 | 5067 | 1.23 | [1.00; 1.52] | 47.6% |
| **Random effects model** | | **2362** | | **6161** | **1.16** | **[1.01; 1.35]** | **100.0%** |

Heterogeneity: $I^2 = 0\%$, $\tau^2 = 0$, $p = 0.81$

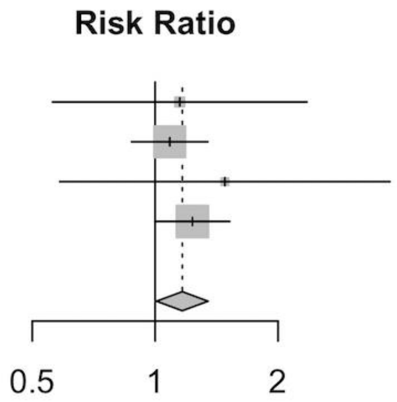

Supplementary Figure S4: Funnel plot for visual inspection of publication bias in studies assessing risk of preeclampsia among young cancer survivors

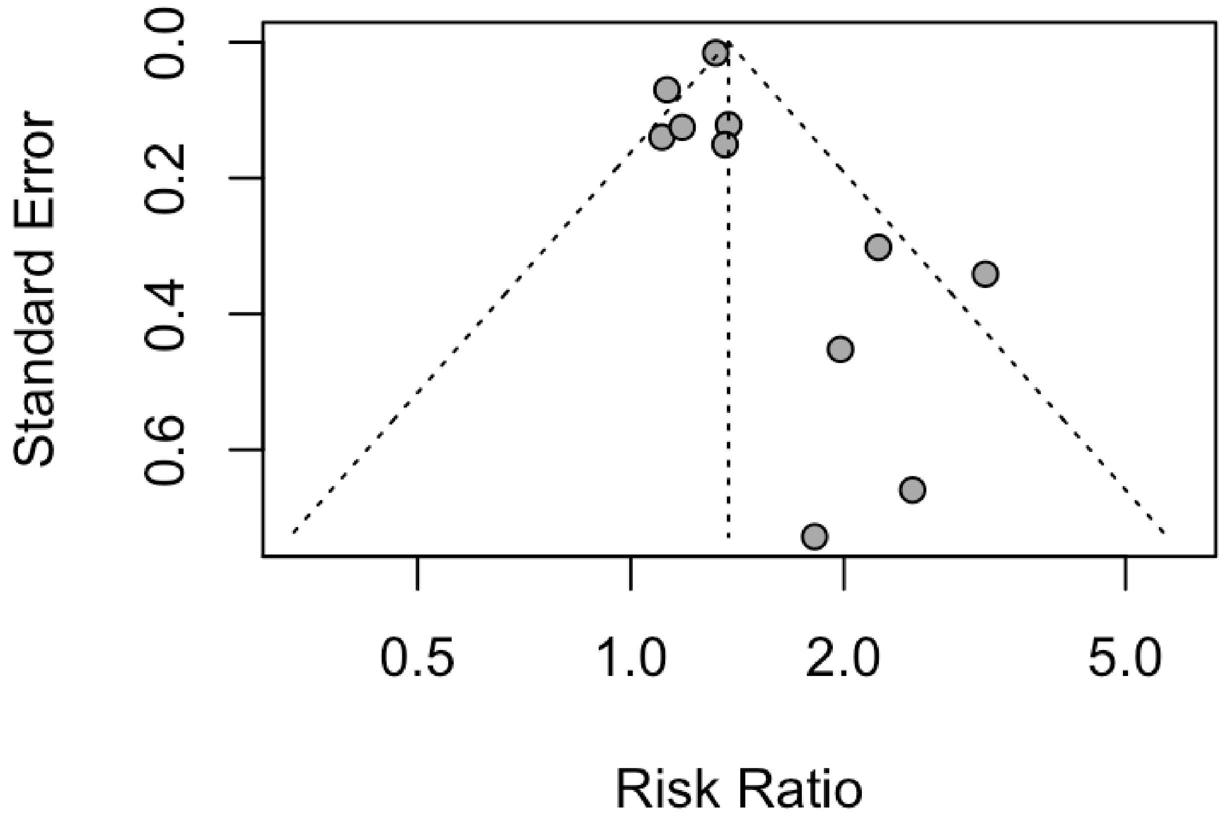

Supplementary Figure S5: Trim-and-fill analysis for publication bias in studies assessing risk of preeclampsia among young cancer survivors

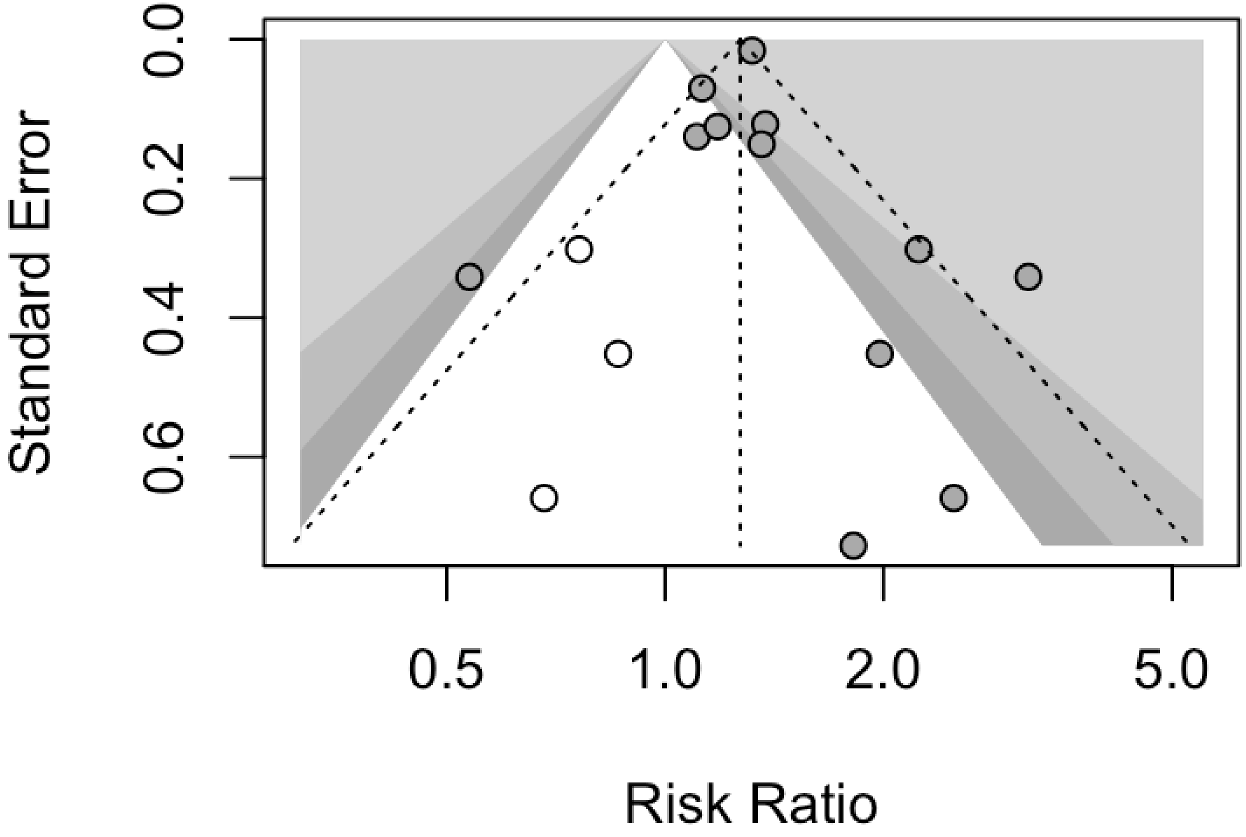

Supplementary Figure S6: Quantitative assessment publication bias in studies assessing risk of preeclampsia among young cancer survivors

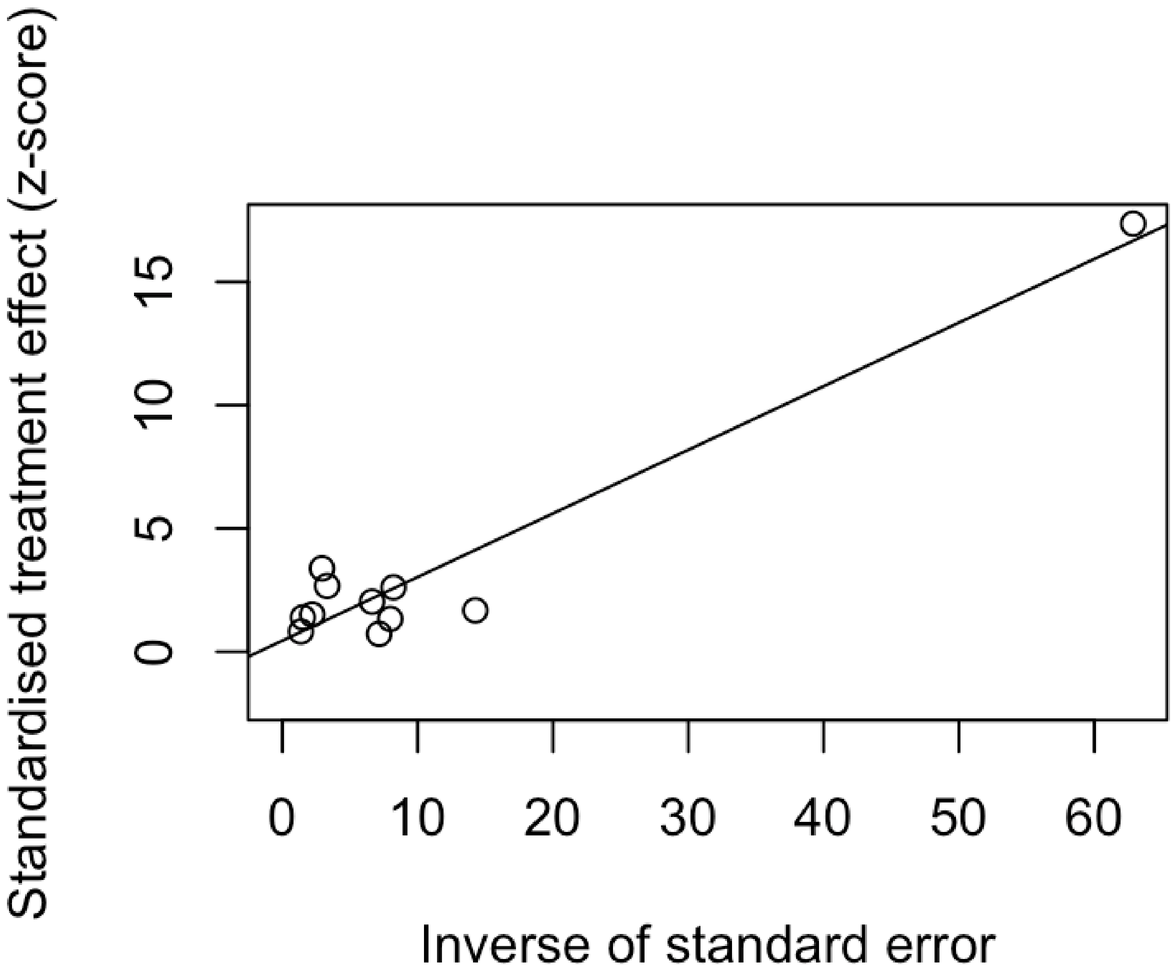

Supplementary Figure S7: Funnel plot for visual inspection of publication bias in studies assessing risk of gestational diabetes among young cancer survivors

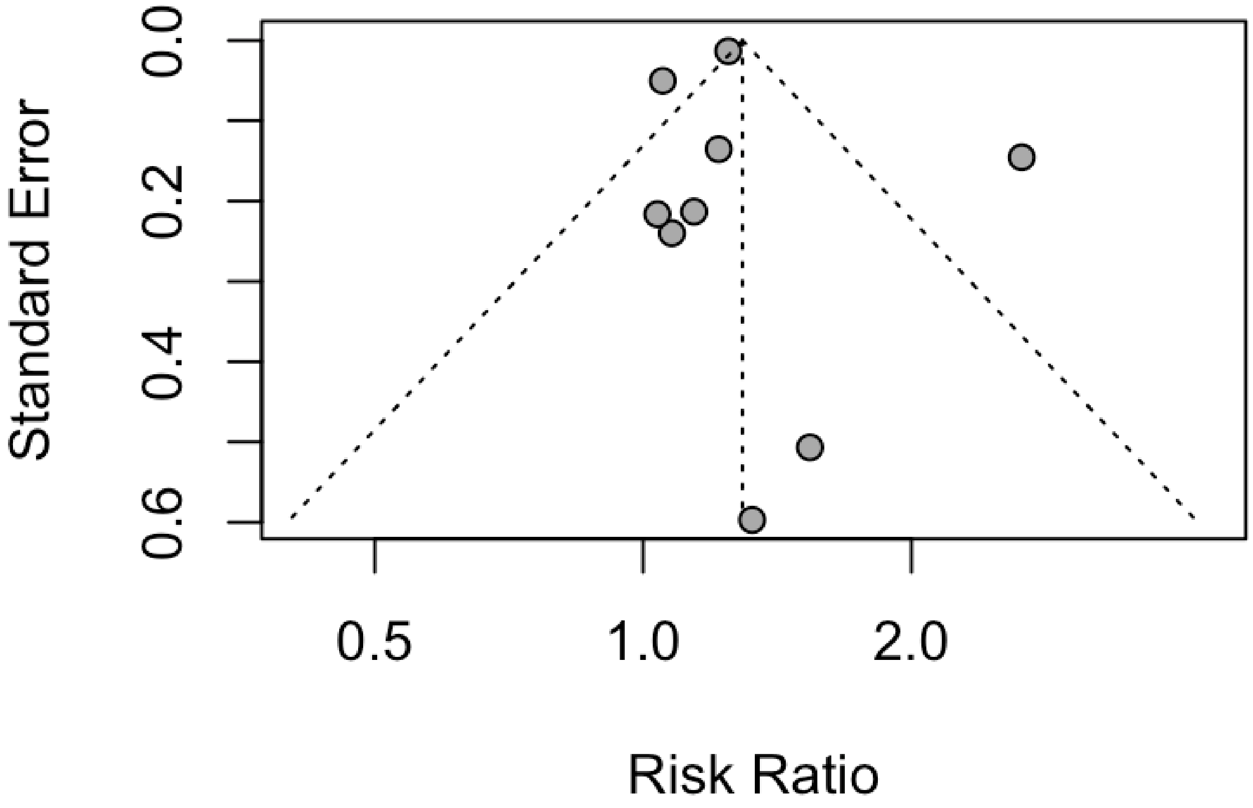

Supplementary Figure S8: Trim-and-fill analysis for publication bias in studies assessing risk of gestational diabetes among young cancer survivors

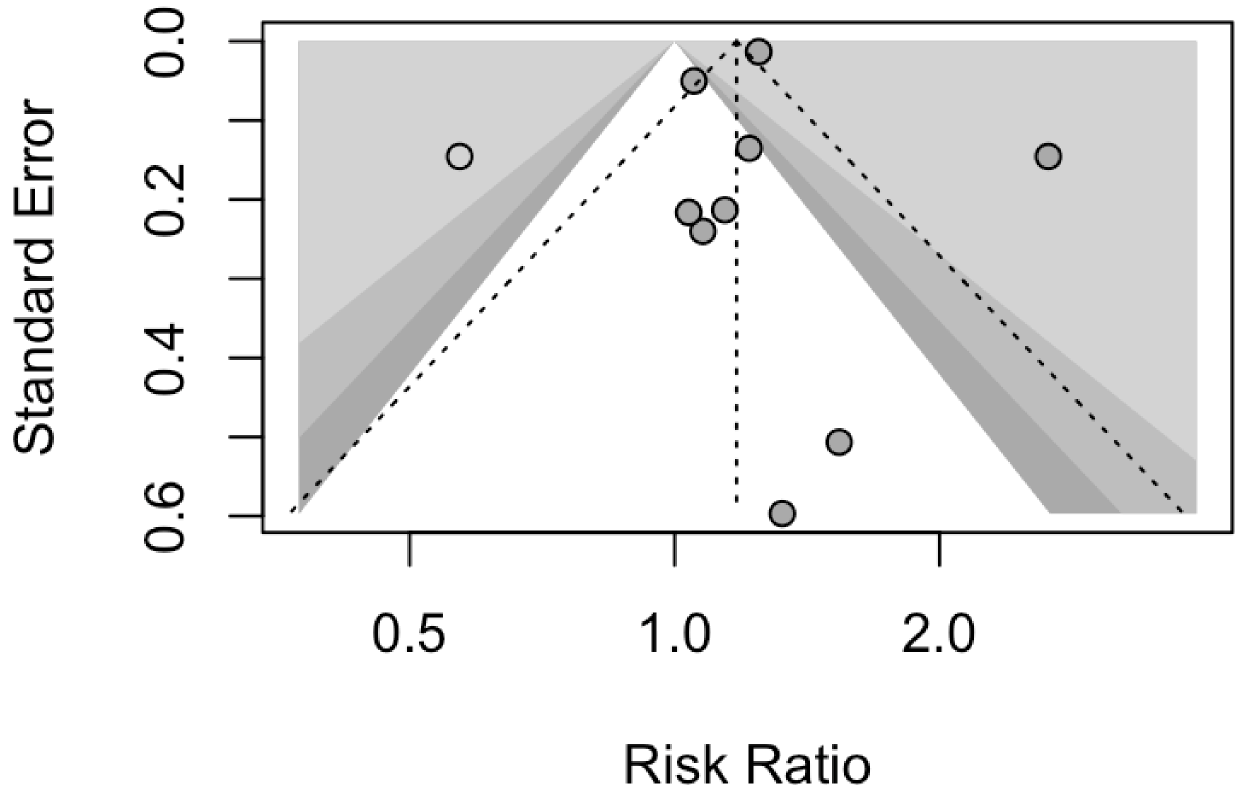

Supplementary Figure S9: Quantitative assessment publication bias in studies assessing risk of gestational diabetes among young cancer survivors

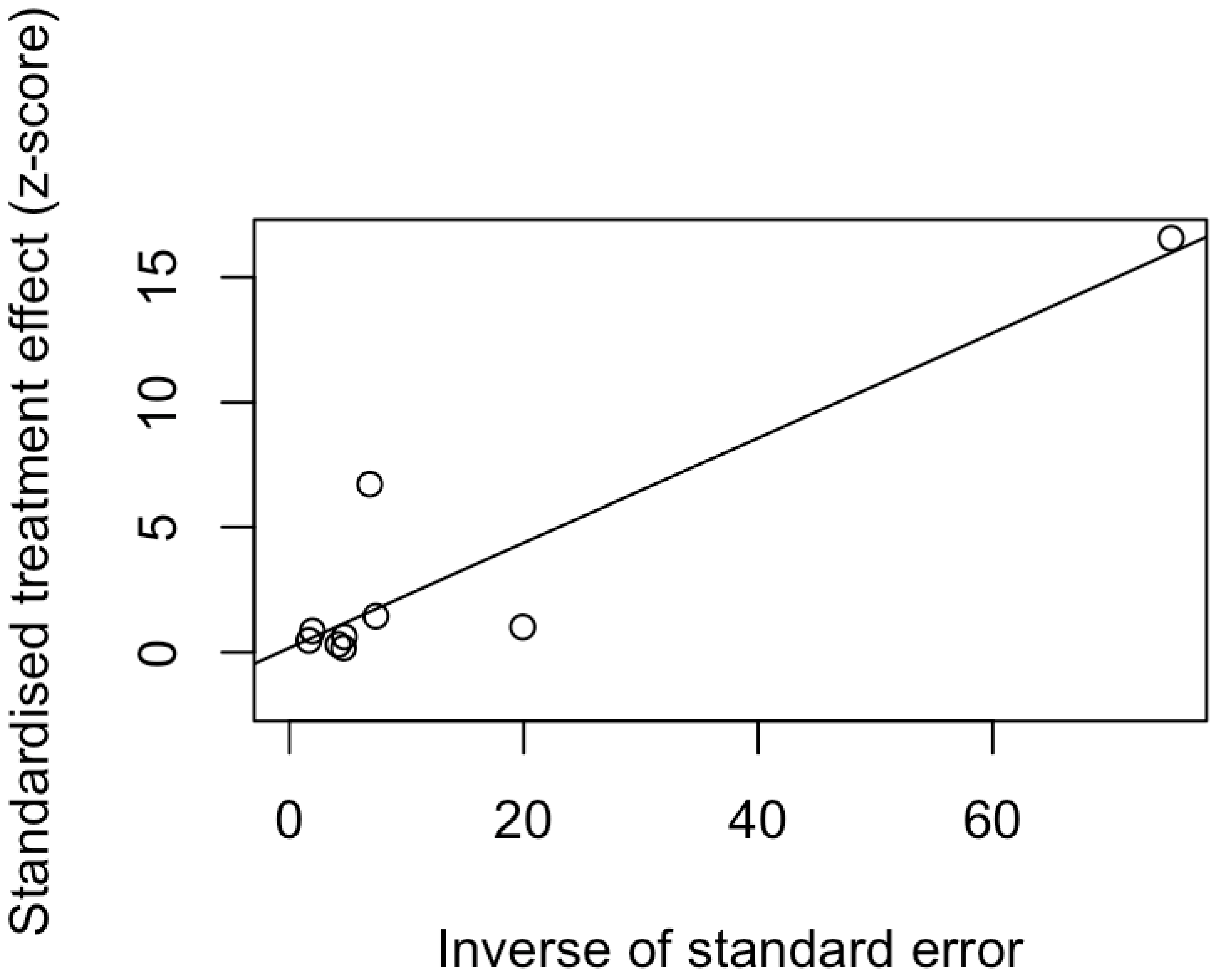

Supplementary Figure S10: Leave-one-out analysis of studies assessing risk of preeclampsia among young cancer survivors, using the random effects model

| Study | RR | 95%-CI | P-value | Tau2 | Tau | I2 |
|---|---|---|---|---|---|---|
| Omitting Cao | 1.37 | [1.15; 1.62] | < 0.01 | 0.0410 | 0.2024 | 50% |
| Omitting Madanat 2010 | 1.37 | [1.15; 1.63] | < 0.01 | 0.0436 | 0.2089 | 51% |
| Omitting Stensheim | 1.40 | [1.15; 1.70] | < 0.01 | 0.0505 | 0.2247 | 52% |
| Omitting Armuand 2019 | 1.28 | [1.17; 1.39] | < 0.01 | 0.0044 | 0.0662 | 27% |
| Omitting Cao | 1.32 | [1.14; 1.53] | < 0.01 | 0.0262 | 0.1620 | 43% |
| Omitting Kao 2023 | 1.43 | [1.19; 1.70] | < 0.01 | 0.0369 | 0.1922 | 36% |
| Omitting Iskender | 1.39 | [1.16; 1.66] | < 0.01 | 0.0488 | 0.2210 | 52% |
| Omitting Niu | 1.42 | [1.19; 1.69] | < 0.01 | 0.0391 | 0.1978 | 49% |
| Omitting Rubens | 1.41 | [1.16; 1.71] | < 0.01 | 0.0495 | 0.2226 | 49% |
| Omitting Haggar | 1.40 | [1.15; 1.70] | < 0.01 | 0.0504 | 0.2245 | 53% |
| Omitting Mueller | 1.42 | [1.18; 1.71] | < 0.01 | 0.0443 | 0.2105 | 51% |
| **Random effects model** | **1.37** | **[1.17; 1.62]** | **< 0.01** | **0.0382** | **0.1956** | **48%** |

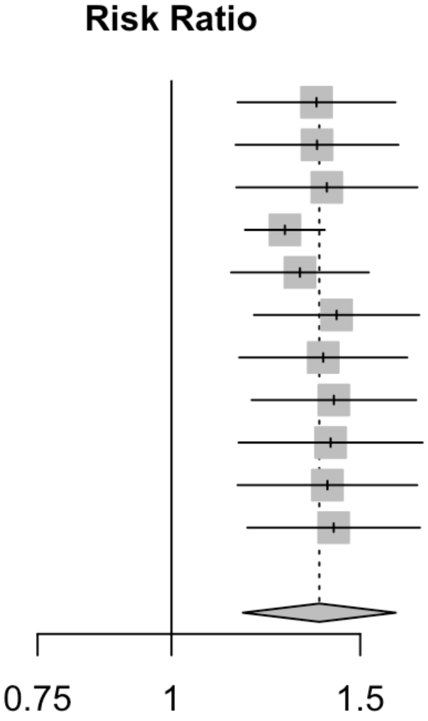

Supplementary Figure S11: Outlier assessment of studies assessing risk of preeclampsia among young cancer survivors, using the random effects model

| Study | Experimental Events | Experimental Total | Control Events | Control Total | RR | 95%-CI | Weight |
|---|---|---|---|---|---|---|---|
| Cao | 5 | 96 | 4 | 192 | 2.50 | [0.69; 9.10] | 0.4% |
| Madanat 2010 | 7 | 1309 | 16 | 5916 | 1.98 | [0.82; 4.80] | 0.9% |
| Stensheim | 65 | 1196 | 3585 | 90632 | 1.37 | [1.08; 1.74] | 10.0% |
| Armuand 2019 | 17 | 278 | 16 | 829 | 3.17 | [1.62; 6.19] | 0.0% |
| Cao | 15 | 207 | 33 | 1019 | 2.24 | [1.24; 4.04] | 2.0% |
| Kao 2023 | 217 | 5151 | 1929 | 51503 | 1.12 | [0.98; 1.29] | 20.6% |
| Iskender | 2 | 18 | 11 | 180 | 1.82 | [0.44; 7.57] | 0.4% |
| Niu | 48 | 707 | 8577 | 139765 | 1.11 | [0.84; 1.45] | 8.0% |
| Rubens | 3741 | 64506 | 822237 | 18687217 | 1.32 | [1.28; 1.36] | 41.2% |
| Haggar | 69 | 1894 | 111 | 4138 | 1.36 | [1.01; 1.83] | 7.0% |
| Mueller | 71 | 1898 | 452 | 14278 | 1.18 | [0.92; 1.51] | 9.5% |
| **Random effects model** | | **77260** | | **18995669** | **1.28** | **[1.17; 1.39]** | **100.0%** |

Heterogeneity: $I^2 = 27\%$, $\tau^2 = 0.0044$, $p = 0.19$

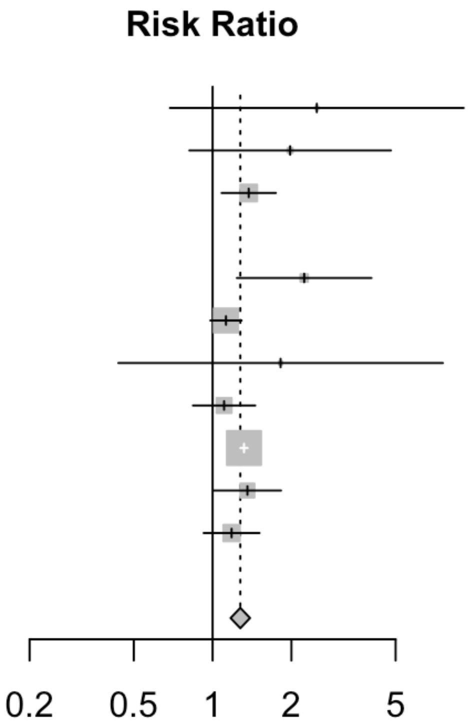

Supplementary Figure S12: Leave-one-out analysis of studies assessing risk of gestational diabetes among young cancer survivors, using the random effects model

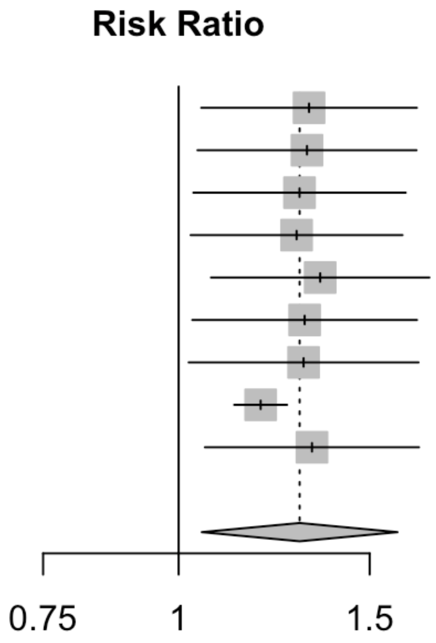


| Study | RR | 95%-CI | P-value | Tau2 | Tau | I2 |
|---|---|---|---|---|---|---|
| Omitting Cao | 1.32 | [1.05; 1.66] | 0.02 | 0.0695 | 0.2637 | 82% |
| Omitting Madanat 2010 | 1.31 | [1.04; 1.66] | 0.02 | 0.0713 | 0.2671 | 82% |
| Omitting Armuand 2019 | 1.29 | [1.03; 1.62] | 0.03 | 0.0734 | 0.2709 | 82% |
| Omitting Cao | 1.28 | [1.03; 1.61] | 0.03 | 0.0721 | 0.2685 | 82% |
| Omitting Kao 2023 | 1.35 | [1.07; 1.70] | 0.01 | 0.0638 | 0.2526 | 75% |
| Omitting Niu | 1.31 | [1.03; 1.66] | 0.03 | 0.0727 | 0.2696 | 82% |
| Omitting Rubens | 1.30 | [1.02; 1.66] | 0.03 | 0.0731 | 0.2703 | 81% |
| Omitting Haggar | 1.19 | [1.13; 1.26] | < 0.01 | 0.0010 | 0.0323 | 41% |
| Omitting Mueller | 1.33 | [1.06; 1.67] | 0.01 | 0.0673 | 0.2595 | 82% |
| **Random effects model** | **1.29** | **[1.05; 1.59]** | **0.02** | **0.0614** | **0.2478** | **80%** |

Supplementary Figure S13: Outlier assessment of studies assessing risk of gestational diabetes among young cancer survivors, using the random effects model

| Study | Experimental Events | Experimental Total | Control Events | Control Total | RR | 95%-CI | Weight |
|---|---|---|---|---|---|---|---|
| Cao | 21 | 96 | 39 | 192 | 1.08 | [0.67; 1.72] | 1.4% |
| Madanat 2010 | 27 | 1309 | 107 | 5916 | 1.14 | [0.75; 1.73] | 1.8% |
| Armuand 2019 | 4 | 278 | 9 | 829 | 1.33 | [0.41; 4.27] | 0.2% |
| Cao | 5 | 207 | 16 | 1019 | 1.54 | [0.57; 4.15] | 0.3% |
| Kao 2023 | 404 | 5151 | 3840 | 51503 | 1.05 | [0.95; 1.16] | 23.0% |
| Niu | 51 | 707 | 8296 | 139765 | 1.22 | [0.93; 1.58] | 4.2% |
| Rubens | 5225 | 64506 | 1214669 | 18687217 | 1.25 | [1.21; 1.28] | 67.3% |
| Haggar | 101 | 1894 | 83 | 4138 | 2.66 | [2.00; 3.54] | 0.0% |
| Mueller | 24 | 1898 | 174 | 14278 | 1.04 | [0.68; 1.59] | 1.7% |
| **Random effects model** | | **76046** | | **18904857** | **1.19** | **[1.13; 1.26]** | **100.0%** |

Heterogeneity: $I^2 = 41\%$, $\tau^2 = 0.0010$, $p = 0.10$

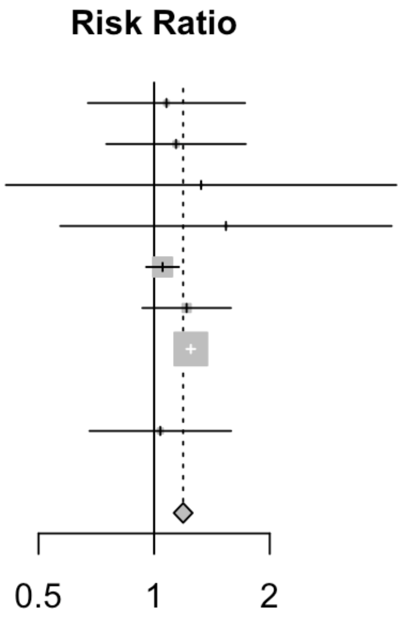

Supplement: Supplementary file 1 [file cancers-17-03924-s001.zip › cancers-3984560-supplementary-proof done.pdf]
